# Recent Development in Nanoconfined Hydrides for Energy Storage

**DOI:** 10.3390/ijms23137111

**Published:** 2022-06-26

**Authors:** Cezar Comanescu

**Affiliations:** 1 National Institute of Materials Physics, Atomistilor 405A, 077125 Magurele, Romania; cezar.comanescu@infim.ro; 2 Department of Inorganic Chemistry, Physical Chemistry and Electrochemistry, Faculty of Chemical Engineering and Biotechnologies, University Politehnica of Bucharest, 1 Polizu St., 011061 Bucharest, Romania; 3 Faculty of Physics, University of Bucharest, Atomiștilor 405, 077125 Magurele, Romania

**Keywords:** hydrogen, energy storage, hydride, nanoconfinement, thermodynamic destabilization, kinetic destabilization, recyclability, MOF, nanomaterials, nanocatalyst

## Abstract

Hydrogen is the ultimate vector for a carbon-free, sustainable green-energy. While being the most promising candidate to serve this purpose, hydrogen inherits a series of characteristics making it particularly difficult to handle, store, transport and use in a safe manner. The researchers’ attention has thus shifted to storing hydrogen in its more manageable forms: the light metal hydrides and related derivatives (ammonia-borane, tetrahydridoborates/borohydrides, tetrahydridoaluminates/alanates or reactive hydride composites). Even then, the thermodynamic and kinetic behavior faces either too high energy barriers or sluggish kinetics (or both), and an efficient tool to overcome these issues is through nanoconfinement. Nanoconfined energy storage materials are the current state-of-the-art approach regarding hydrogen storage field, and the current review aims to summarize the most recent progress in this intriguing field. The latest reviews concerning H_2_ production and storage are discussed, and the shift from bulk to nanomaterials is described in the context of physical and chemical aspects of nanoconfinement effects in the obtained nanocomposites. The types of hosts used for hydrogen materials are divided in classes of substances, the mean of hydride inclusion in said hosts and the classes of hydrogen storage materials are presented with their most recent trends and future prospects.

## 1. Introduction

The 21st century has been marked by tremendously important technological breakthroughs, yet the massive expansion of industrialization has led to a deepening scarcity and skyrocketing prices of fossil fuels and energy raw materials, concomitant with a continual atmospheric pollution [1]. In the context of ever-increasing energy demands and the serious downsides of using fossil fuels, hydrogen has emerged over the past decades as a true and relevant promise of a carbon-free, green energy source for the world. However, hydrogen has a very low boiling point (20.4 K) at 1 atm, which severely restricts its use in the native form, except in some high pressure, cryogenic tanks that pose themselves additional energetic costs and safety risks regarding charging, transport and storing [1]. To circumvent the downfalls of using molecular dihydrogen (H_2_), scientists have turned their attention and research focus on hydrogen-containing compounds, in the form of metal hydrides and related materials, which in turn feature higher thermal stability, safer handling, no fuel loss upon storage and overall produce the cleanest energy known today. The fuel of the future should ideally produce no carbon-containing by-products, exhibiting time- and property-related endurance over 1500 dehydrogenation-rehydrogenation cycles, and most importantly, all of this while featuring a gravimetric weight percentage of at least 5.5 wt.% (DOE’s target set for 2025) [1,2,3,4,5,6]. The use of fossil fuels will eventually be phased-out and an energy-friendly alternative with no carbon emissions must be brought forward. Hydrogen can generate roughly three times more energy than gasoline (33.3 vs. 11.1 kWh/kg), and can be produced by thermochemical, electrolytic, solar splitting of water or by means of biological reactions involving bacteria and algae microorganisms [1]. The near-future (2025) targets for hydrogen storage systems require a system gravimetric capacity of 5.5 wt.%, volumetric capacity 0.04 kg H_2_/L, a hydrogen delivery temperature range of −40…+85 °C, a delivery pressure of five bar, a very fast refill time (3–5 min) and a high purity hydrogen production of 99.97% [4,5,6]. Broadly speaking, the hydrogen storage methods are divided in physisorption-based (fast kinetics, storage capacity dependent on support surface area and pore volume, weak Van der Waals interatomic forces) and chemisorption-based (somewhat slower kinetics of desorption/absorption, storage depends on chemical composition of the material, strong chemical bonds) [7,8]. The chemisorption method is the preferred one of the two, as it binds hydrogen through chemical bonds rather than weaker interatomic forces therefore ensuring a reliable hydrogen storage capacity and comprise of metal hydrides, tetrahydridoborates, tetrahydridoaluminates and metal amides.

The topic of hydrogen storage materials has been recently reviewed by a series of articles. Lai et al., have summed up the characteristics of potential hydrogen storage materials and established guidelines that new storage materials should obey for viable applicability in storage tanks [9]. An overview of hydrogen economy and appropriate recommendations was discussed by Abe et al. [10]. The state-of-the-art of boron-nitrogen compounds for energy storage was reviewed by Kumar et al. [11] and Hagemann [12]. The solid-state materials used for hydrogen storage have been addressed by Lee et al. [13], Hadjixenophontos et al. [14], Broom and Hirscher [15], Comanescu [16], Kharbachi et al. [17], Zheng et al. [18] and He et al. [19], among others. The role of highly dispersed catalysts on hydrogen storage materials [20] and the topologically engineered materials serving for energy conversion and storage [21] have also been very recently reviewed, while the critical issue of accurately describing hydrogen sorption properties of materials has been highlighted by Broom et al. [15].

Among hydrogen storage materials, metal hydrides have gained increased popularity [5,6,7,8,22,23,24,25,26] and channeled many research groups to corroborate hydrogen sorption data to formulate general design principles for these materials [27,28], or to tackle the imminent need to expand current knowledge to production of large-scale hydrogen storage facilities [29]. While many advances have been made in the field of metal hydrides for hydrogen storage applications, the high thermal stability, sluggish kinetics and poor reversibility of hydrogen release/uptake have shifted researchers’ attention towards nanoconfined hydrides that seem to alleviate some of these drawbacks, affording reversible, high gravimetric and volumetric hydrogen content at moderate temperatures [8,24,25].

The current review aims to tackle the current trend of employing nanoconfinements as a reliable tool to tune kinetic and thermodynamic behavior of hydride materials used for energy storage applications, and covers roughly the past five years.

## 2. Characterization Methods: Old, New, and Their Pitfalls

Traditionally, hydrogen storage materials follow a typical characterization protocol involving structural (XRD), elemental (XPS), morphological (SEM, TEM, N_2_ sorption isotherms) and recording of hydrogenation data (PCI curves) [8]. Recently, a fundamental issue regarding elucidation of local environment of hydrogen in energy materials has revealed fast sample spinning ^1^H NMR high-resolution spectroscopy as an appropriate tool to quantitatively characterize hydrogenated TiZrNi quasicrystals [30]. Kweon et al., showed by employing fast-spinning NMR spectroscopy that neutral hydrogen is surrounded by metal atoms shifting gradually from Zr to Ti and then Ni with increasing hydrogen content [30]. ^1^H magic-angle spinning (MAS) NMR spectra has shown real promise for tuning electronic characteristics in a Ba-Ti oxyhydride, and could become a tool to investigate hydrogen occupation in the vicinity of the nuclei (negative Knight shift, indicative of interaction of conduction band electrons and probe nucleus) [31]. A potential downside of using this technique is the high sensitivity to sample temperature, which was shown to increase due to fast rotor spinning (10–35 Hz), with a direct effect on main peak width change. Thus, additional precautions need to be undertaken to account for the effect of sample temperature increase when using fast spinning NMR spectroscopy [31]. 

Correct understanding of interfacial phenomena occurring during hydrogen storage is now termed as hydrogen spillover effect (HSPE). First discovered in 1964, it describes the migration of hydrogen atoms produced by H_2_ decomposition on an active site, and it allows for a more insightful view on the dynamic behavior of hydrogen in energy storage materials [7]. While molecular orbital energy computations showed unfavorable energy for H atom spillover on non-reducible supports, recent studies have shown that HSPE is indeed possible on inert supports such as siloxanic materials (SiO_2_) [7]. This bears a direct effect on hydrogen storage materials such as metal hydrides confined in mesoporous silica supports, where the spillover distance is limited to very short distances of ~10 nm [7].

**Figure 1:**
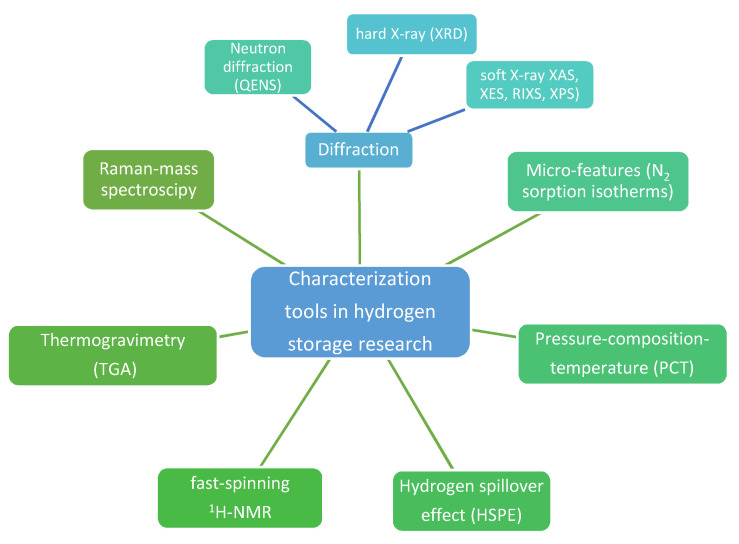
Main investigation methods used for characterization of hydrogen storage materials.

Interestingly, developing tools to characterize metal hydrides during hydrogenation cycles has led to a summary of soft (X-ray absorption, XAS; X-ray emission spectroscopy, XES; resonant inelastic soft X-ray scattering, RIXS, X-ray photoelectron spectroscopy, XPS) and hard (X-ray diffraction, XRD) X-ray techniques used to this end (Figure 1) [32]. Soft X-rat techniques (100–5000 eV) are particularly appealing for tracking mechanistic behavior and intermediate product formation during hydrogenation studies, with direct influence over hydrogen storage capacity. XAS measurements for instance are bulk or surface-sensitive, and show 3d transition metal (TM) L-edges corresponding to transition of a 2p electron to an unoccupied 3d orbital, hence enabling monitoring of oxidation state changes during hydrogen release (+n...0) and uptake (0…+n) [32]. Similarly, TM-catalyzed alanates (2 mol%-catalyzed NaAlH_4_) showed in XAS measurements the Al and Na K-edge and Ti L-edge consistent with a Ti-like state throughout the hydrogen release/uptake cycles, but with clear differences in Al state, which may undergo various intermediate states (Al/NaAlH_4_/Na_3_AlH_6_) [32]. Quasi-elastic neutron scattering (QENS) studies have been undertaken to establish hydrogen dynamics in nanoscale sodium alanate NaAlH_4_ and showed that fitting QENS to a Lorentzian function can yield two dynamic states of hydrogen and concluded that even at 77 °C there is a high percentage (18%) of mobile hydrogen atoms in the nano-NaAlH_4_ [33].

As an alternative method to the conventional pressure-composition-temperature (PCT) method typically used to characterize thermodynamic parameters for hydride-based systems, a less complex investigation method has been described for MgH_2_-based materials: thermogravimetric analysis (TGA) [34]. This method relies on cycling the hydride under a flowing gas of constant hydrogen partial pressure, and the TGA curves are further analyzed using the van’t Hoff equation to obtain the absorption/desorption enthalpies, which in the case of VTiCr-catalyzed Mg/MgH_2_ materials, showed good agreement with traditional PCT results [34]. Other recent research established a nano-Pd patched surface of Pd_80_Co_20_ to afford one of the most sensitive optical hydrogen sensors (fast response of <3 s, high accuracy of <5%, and very low limit of detection of 2.5 ppm) [35]. Employing interpretable machine learning could also help formulate general design principles for intermetallic hydride-based systems being used to validate limited data from the HydPARK experimental metal hydride database and stressing the recommendation for experimental groups to report ΔH, ΔS, P_eq_, T and V_cell_ [27]. 

Valero-Pedraza et al., have characterized the hydrogen release form ammonia borane nanoconfined in mesoporous silica by means of Raman-mass spectroscopy, which confirmed hydrogen release from AB at lower temperatures, fewer BNHx gaseous fragments in nanoconfined samples and a lack of polyiminoborane formation during thermolysis [36]. The study also pointed out to silica-hydride interactions, which were identifiable based on modifications in the Raman spectra [36]. 

However, analysis of the literature data also points out to several weaknesses in applying traditional characterization methods that have not yet been tuned for current nanosized materials [15,34,37,38]. For instance, AB (ammonia borane) hydrogenation studies showed many inconsistencies [38]. By assessing TGA data in the literature, Petit and Demirci urge caution when evaluating ammonia borane weight loss (and consequently hydrogen release), as this was found to be highly dependent on the operation conditions (semi-closed/open reactor) and were shown to erroneously indicate a different hydrogen release temperature onset and hydrogen wt.% [38].

Surrey et al., conducted a critical review of a paper discussing electron microscopy observation of elementary steps in MgH_2_ release mechanisms [37]. In this work, they debunked the general assumption that TEM microscopy can be used, as such, without further testing methodology adjustment in the case of hydrogen storage materials such as MgH_2_. The issue was serious, as it led initial authors to misinterpret TEM observations, by disregarding the key aspect of electron beam induced dehydrogenation of MgH_2_ [37]. In a cascade chain of errors, the beam-induced heat producing dehydrogenation also led to a false interpretation of SAD (selected area diffraction) data, which only showed hollow MgO shells deprived of Mg-core, an effect actually ascribed to the nanoscale Kirkendall effect. As a result, it was apparent that the sample actually measured did not even contain MgH_2_ any longer [37]. 

In line with the issues raised above, Broom and Hirscher discussed the necessary steps for reproducible results in hydrogen storage research [15]. 

## 3. Bulk vs. Nanomaterials

**Figure 2:**
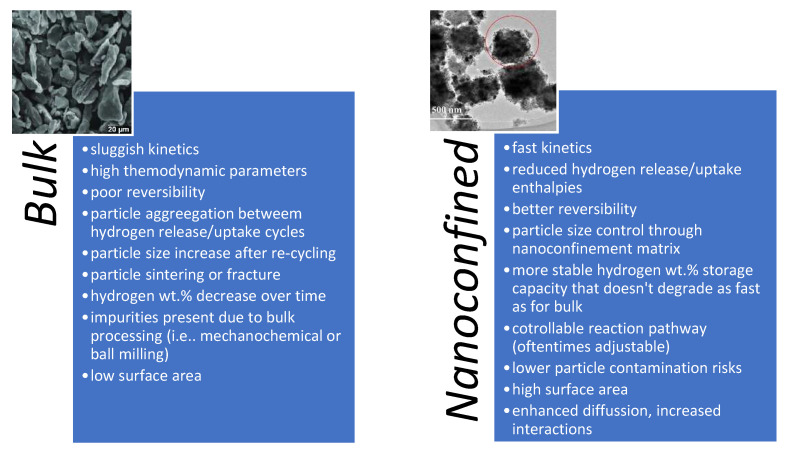
Main features of bulk and nanoconfined materials for hydrogen storage; exemplified for the case of an overly-studied hydride, MgH_2_. (inset reprinted/adapted with permission from Ref. [65]. 2022, Elsevier).

After its first inclusion on the research outlook of scientists worldwide in 1996, nano-sized hydrides have known a wide expansion, mainly due to several important kinetic and thermodynamic improvements of nanoconfinement over their bulk counterparts [4,8,14,16,18,21,22,23,27,28,39,40,41,42,43,44,45,46,47,48,49,50,51,52,53,54,55,56,57,58,59,60,61,62,63,64]. Over time, nanoconfinement has emerged as a reliable tool for tuning not only thermodynamic and kinetic behavior at nanoscale, but also for altering reaction pathways, lowering or even suppressing side-reactions and side-products, while also affording better size control of the particles over several hydrogen release/uptake cycles (Figure 2).

### 3.1. Physical and Chemical Aspects of Nanoconfinement Effects

Nanoconfinement of active hydride species inside a porous host bears a number of physical and chemical implications [1,7,8,22,25,27,41,52,53,54,55,56,59,60,61,62,66,67,68,69,70,71,72,73,74,75,76,77,78,79,80,81,82,83,84,85,86]. 

### 3.2. Nanocomposites

The proper term for describing the materials resulting from the nanoconfinement of active hydride source into a nanoporous matrix is nanocomposite [4,41,52,54,60,66,68,69,70,77,78]. 

## 4. Types of Hosts Used as Hydride Matrix

### 4.1. Siloxanic Materials (MCM-41, SBA-15, SBA-48, etc.)

Although some complex hydride materials (e.g., complex metal borohydrides) are plagued by an undesirable reaction with the porous host above the hydride melting temperature with formation of silicates [16], mesoporous silica is still used in several studies concerning nanoconfinement effects in hydrogen storage materials [74,87,88,89,90] (Table 1). 

When LiBH_4_ was used as borohydride source in a mesoporous silica host, the reaction occurring during borohydride melting is a two-step process yielding lithium metasilicate, Li_2_SiO_3_, and ultimately lithium orthosilicate, Li_4_SiO_4_ (Equation (1)) [16]. This reaction is a downside of using nanoporous siloxanic supports for borohydride nanoconfinement, as it consumes the hydride material in an irreversible side-reaction (Equation (1)).

(1) $$4{\mathrm{LiBH}}_{4\;}+3{\mathrm{SiO}}_{2}\overset{\Delta }{\Rightarrow }\;2{\mathrm{Li}}_{2}{\mathrm{SiO}}_{3}+\mathrm{Si}+4B+8H_{2\;}\overset{2{\;\mathrm{LiBH}}_{4}}{\Rightarrow }\;\frac{3}{2}{\mathrm{Li}}_{4}{\mathrm{SiO}}_{4}+\frac{1}{2}\mathrm{Si}\;+2B+4H_{2}↑\;$$

Confining LiBH_4_ by a melt impregnation technique in nanoporous silica MCM-41 (1D, d_pore_ < 2 nm) or SBA-15 (2D-ordered pore structure, d_pore_ = 5, 7 and 8 nm) of different pore sizes reveals an interesting interfacial effect governing Li^+^ and BH_4_^−^ ion mobility [87]. Using solid-state NMR (^1^H, ^6^Li, ^7^Li and ^11^B), Lambregts et al., showed that, as a result of nanoconfinement, two distinct fractions of LiBH_4_ coexist and this is a temperature-dependent equilibrium (Equation (2)):

(2) $${\mathrm{LiBH}}_{4\left(\mathrm{bulk}-\mathrm{like}\right)}\overset{T}{\Leftrightarrow }{\;\mathrm{LiBH}}_{4\left(\mathrm{highly}\;\mathrm{dynamic}\right)}\overset{T}{\Leftrightarrow }{\;\mathrm{LiBH}}_{4\left(\mathrm{lower}\;\mathrm{mobility}\right)}$$

The high mobility LiBH_4_ is located near silica pore walls, whereas LiBH_4_ of lower mobility is located towards the pore’s core; the theoretical wall thickness was estimated based on a core-shell model LiBH_4_@SBA-15, as $t=r_{p}(1-\sqrt{f_{lower\;mobility}}$. The dynamic layer thickness is temperature-dependent, and increases from 0.5 nm (30 °C) to 1.2 nm (110 °C). Here again the results of calorimetric data were found to overestimate the highly-mobile LiBH_4_ layer thickness (1.9 nm), pointing out the need for care when deriving the same parameter from different techniques [87]. While ^6,7^Li NMR spectra was too complex for unequivocal deconvolution, ^1^H and ^11^B NMR spectra clearly show two components throughout the investigated temperature range (30–130 °C), consistent with the two LiBH_4_ fractions of different ion mobility [87].

**Figure 3:**
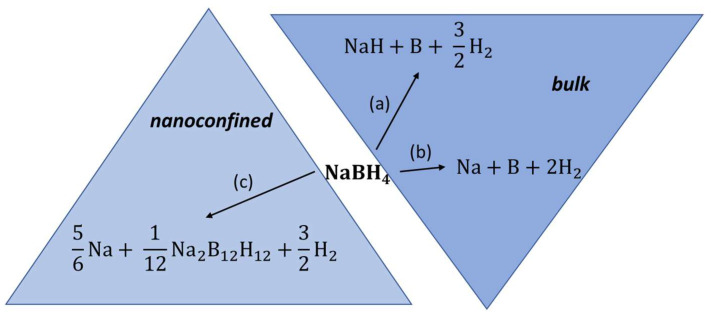
Possible decomposition pathways for bulk NaBH_4_ (a,b) and for melt-impregnated, nanoconfined NaBH_4_ (c).

Melt impregnation of NaBH_4_ in MCM-41 at 560 °C led to a drastic surface area decrease from 1110.9 m^2^ g^−1^ (pristine MCM-41) to 3.5 m^2^ g^−1^ (nanocomposite NaBH_4_@MCM-41), and to a 78% pore filling attested by pore volume decrease (1.02 cm^3^ g^−1^ to 0.02 cm^3^ g^−1^) [74]. Interestingly, some amount of sodium perborate NaBO_4_ resulting from unavoidable oxidation of the borohydride with silanol (Si-OH) groups is the main additional phase detected by XRD, confirming no significant additional phases due to melt impregnation at >500 °C. The dehydrogenation onset peak for NaBH_4_ was reduced by nanoconfinement from 550 °C (bulk) to 520 °C (nanocomposite) [74]. Due to the insulating nature of boron oxide phase (NaBO_4_), the ionic conductivity did not improve the same way it does for LiBH_4_, and remained largely the same (7.4 × 10^−10^ S cm^−1^). This 10-fold increase in ionic conductivity that only lasts up to 70 °C for the nanocomposite is attributed to the presence of larger dodecaborate ions B_12_H_12_^2−^ whose distinct presence was signaled in ^11^B NMR spectra by an additional sharp peak at −15.58 ppm (NaBH_4_@MCM-41) vs. −41.95 ppm (for pristine BH_4_-) (Figure 3) [74].

The organic–inorganic hybrid poly(acryalamide)-grafted mesoporous silica nanoparticles (PAM-MSN) have been evaluated as functionalized nanoporous hosts for tuning hydrogen release/uptake behavior in ammonia borane (AB), which started to desorb hydrogen in the said nanocomposite at a lower temperature with respect to pristine AB, which was further enhanced by functionalization of the mesoporous silica shell with carboxylic -COOH groups [88]. 

2D-ordered mesoporous silica of cylindrical pores (SBA-15) was successfully used by Yang et al., for enhancing the ionic conductivity of a mixed-anion borohydride, Li_2_(BH_4_)(NH_2_). By following a melt infiltration procedure, the Li-ion conductivity was increased in Li_2_(BH_4_)(NH_2_)@SBA-15 to 5 × 10^−3^ S cm^−1^ at 55 °C [89]. A marked kinetic improvement of hydrogen release (ΔT = 70 °C) was recently reported by Rueda et al., by confinement of ammonia borane (AB) in silica aerogel by simultaneous aerogel drying and AB gas antisolvent precipitation using compressed CO_2_, and achieving a weight AB loading of up to 60 wt.% [90].

### 4.2. Carbonaceous Materials (C-Replica of Mesoporous Silica, C-NTs, C-Foam, C-Spheres, Graphene, Graphene Oxide GO, Reduced Graphene Oxide r-GO)

Given the chemically-sensitive interaction between silanol (Si-OH) and borohydride (BH_4_^-^) groups and the subsequent oxidation reaction, the election of mesoporous silica as a host for loading borohydride materials seems less feasible. Therefore, many research studies have shifted their focus towards carbon-based materials, which do not exhibit such a drawback. Many forms of carbonaceous matrix have been employed: C-replica of mesoporous silica, C-NTs, C-foam, C-spheres, graphene, graphene oxide GO, reduced graphene oxide r-GO etc. (Table 2) [40,42,53,65,69,70,92,93,94,95,96,97,98,99,100,101,102,103,104,105,106,107,108,109,110,111,112,113,114,115,116,117,118,119,120,121,122,123,124,125,126,127,128,129,130,131,132,133,134,135,136,137,138,139,140,141,142,143,144,145,146]. 

### 4.3. Metal-Organic Frameworks (MOFs) and Functionalized-MOFs

Metal-organic frameworks (MOFs) have recently been utilized as hosts for metal hydrides, due to their tunable porosity, stability and enhancement of kinetic and thermodynamic properties of hydrogen storage materials (Table 3). Their functionalization with appropriate groups/molecules opens new doors in energy storage field, being able to bypass side-reactions, alter significantly the reaction pathway, and afford a better reversible material in hydrogen release/uptake studies [39,40,68,86,147,148,149,150,151,152,153,154,155,156,157].

### 4.4. Main Group and TM (Transition Metal)-Oxides, Sulfides and Nitrides

Various metal oxides and nitrides of metals (main group and TM) have been employed as hosts for hydrogen storage materials [91,95,101,106,147,158,159,160,161,162,163,164,165,166,167,168,169,170,171,172]. Embedding active hydrogen-storage systems into inert nanoscaffolds has been used in the past, but reports on shells of the sulfide type are rare in the scientific literature [80]. In fact, the only recent report is that of MgH_2_ nanoconfined in chemically-inert shells of CoS nano-boxes [80] (Table 4). 

### 4.5. Metal Component/Host

Several reports have been published where the host is an actual metal matrix, usually one that is highly active in hydrogenation studies (Table 5) [40,68,94,112,121,122,138,163,164,165,166,167,168,169,170,171].

### 4.6. Gas Selective-Permeable Polymers

Attempts to restrict oxygen and moisture exposure of active hydrogenation sites in hydride materials have been made through the engineered approach of covering the hydride materials with a layer of H_2_-permeable polymer [88,127,156,173,174,175]. This approach proved to be very successful, provided that the hydride coverage was indeed complete (Table 6).

### 4.7. MXene

Ongoing recent trends in developing novel systems for energy storage have incorporated MXene materials with a 2D structure, as promising hydride hosts [94,156,176,177,178,179,180,181,182,183,184,185,186,187,188,189,190,191,192,193,194]. While only few examples are currently available, it is foreseeable that MXenes will grow to become a mainstream storage matrix for nanoconfined hydride-based materials (Table 7).

### 4.8. Catalytic Effects of Doping the Host and/or Substitution of the Hydride Species

Improvements on hydrogen release/uptake cycles have often been explored in conjunction with utilization of catalysts used to either dope the host, or the hydride material. This strategy is based on formation of active sites for hydrogenation reaction to occur, or is sometimes ascribed to the formation of a reactive intermediate species [19,68,92,102,111,112,113,117,125,128,151,160,161,163,195,196,197]. In addition, cation substitution or anion substitution in complex hydrides has been employed to reduce energy barriers and improve overall recyclability of the hydride materials (Table 8).

### 4.9. (Nano)Catalyst Addition

The overall enhancement of kinetic and thermodynamic parameters can be tuned by utilization of catalysts. This is usually implemented to improve behavior of systems that already show promising results including recyclability (Table 9) [19,20,34,43,57,65,68,77,82,92,102,108,113,118,120,125,131,132,134,136,139,143,147,158,160,166,167,168,172,183,184,185,186,187,188,189,190,191,192,194,196,197,198,199,200,201,202,203,204,205,206,207,208,209,210,211,212]. Due to the greater applicability of this approach in the past few years, the Table 9 summarizes them based on classes of substances and their corresponding characteristics. 

## 5. Inclusion Methods of Hydride Materials into Appropriate Host—State-of-the-Art and Limitations

### 5.1. Direct Synthesis

Starting from a commercially-available borohydride (such as LiBH_4_, NaBH_4_ etc.) and the corresponding salt of the metal (MCl_x_), various novel borohydrides have been synthesized via the metathesis reaction (double exchange) (Equation (3)) [8,16].

(3) $${\mathrm{MCl}}_{x}+{x\;\mathrm{LiBH}}_{4}\Rightarrow M{\left({\mathrm{BH}}_{4}\right)}_{x}+\;x\;\mathrm{LiCl}\;$$

Other approaches start from the organometallic precursor of the metal, which undergoes reduction (with H_2_ or another reductant, such as LiNp) typically after impregnation into the porous host. (Equation (4))

(4) $$\mathrm{Mg}{\left(C_{4}H_{9}\right)}_{2}+2{\;H}_{2}\;\overset{\Delta }{\Rightarrow }{\;\mathrm{MgH}}_{2}+2{\;C}_{4}H_{10}$$

### 5.2. Infiltration Methods

#### 5.2.1. Melt Infiltration

Melt infiltration of complex hydrides has widely been used to introduce the active hydride material into nanoporous hosts. This technique has the advantage of requiring no solvent (so it consists of less steps), but the hydride material must have a lower melting temperature, and the infiltration is carried out under H_2_ pressure in order to avoid the onset of dehydrogenation reaction.

#### 5.2.2. Solvent Infiltration

Solvent infiltration has become the method of choice as it achieves pore filling of the porous scaffold at temperatures that are near ambient, provided that a suitable solvent for the material has been identified. This is typically an issue, as solubility data on complex hydrides are rather scarce, and usually their solubility in ether-like solvents is limited [16].

#### 5.2.3. Solvent-Assisted Ball-Milling

Nanoconfinement of hydride-based materials in nanoporous hosts has the potential advantage of bypassing the slow kinetics of their bulk counterparts, thus enabling a shorter refueling time, in pursuit of the DOE’s current targets [5,6]. Very high surface area supports (MOFs, activated carbons) afford good hydrogen sorption capacities, but since the adsorption is mainly governed by physisorption, it is only relevant at 77 K. At this low temperature, a rough estimation (Chahine’s rule) is that for pressures that would occupy all adsorption sites (exceeding 20 bar), the expected storage capacity is ~1 wt.%/500 m^2^g^−1^ and scales proportional to the specific surface area [8]. Ball milling (with or without a solvent) can introduce the hydride material into the porosity of the employed scaffold. The process is energy-intensive and can proceed with an important increase in the local sample temperature, and therefore the process is carried out in steps (for instance, 20 min milling followed by a 10 min pause allowing controlled cooling). 

## 6. Metal Hydrides and Their Recent Nanoconfinement Studies

Pristine metal hydrides have recently been comprehensively reviewed, and the results show promising trends upon nanoconfinement [213].

### 6.1. LiH

Alkali metal hydrides have been used for catalytic reactions, but have attracted attention due to their lightweight characteristics, as well as the high hydrogen gravimetric content. However, their high thermal stability makes them less attractive in their pure form; LiH, for instance, melts at 689 °C and decomposes at 720 °C into Li and H_2_ (Equation (5)). Alkali metal hydrides have unusually high decomposition temperatures due to their salt-like nature (LiH, mp = 698 °C; NaH, mp = 638 °C; KH, mp ~ 400 °C with K vaporizing in H_2_ current). Given their high decomposition temperature, alkali metal hydrides require kinetic and thermodynamic destabilization (Table 10).

(5) $$\mathrm{LiH}\overset{720\;{}^{\circ}C}{\Rightarrow }\mathrm{Li}+\frac{1}{2}H_{2\;}$$

Recently, a series of strategies have been utilized to produce nanosized LiH, but not all attempts dealt with hydrogen storage applications [114,133,198,214,215,216], and some utilizing LiH-containing nanocomposites for their Li-storage capacity in a Co(OH)_2_-LiH novel anode material [217]. Even when dealing with potential hydrogen storage materials like LiH + MgB_2_, studies have focused on the phase-evolution process and XPS tracking thereof, rather than collection of hydrogen storage data [198]. Still, XPS data pointed to presence of LiBH_4_, Mg_(3−x)/2_Li_x_(BH_4_)_x_ or Li-borate species present on account of multiple LiH-containing peaks identified [198]. At near-surface regions, LiBH_4_ or mixed Li-Mg borohydrides can form at 100 °C below the threshold for hydrogenation of MgB_2_; expectedly, LiBH_4_ production scales with the LiH in the starting composite (Equation (6)) [198].

(6) $$\mathrm{LiH}+\frac{1}{2}{\mathrm{MgB}}_{2}+2H_{2}\overset{\Delta }{\Rightarrow }{\mathrm{LiBH}}_{4}+{\mathrm{MgH}}_{2}$$

Sun et al., have shown that harnessing the plasmonic thermal heating effect of Au nanoparticles could lead to light-induced dehydrogenation of nanocomposites Au@LiH, which showed a 3.4 wt.% loss ascribed to dehydrogenation content [214]. The Au NPs dispersed on the surface of LiH, Mg or NaAlH_4_ all showed marked improvements in hydrogenation studies. The preparation of Au/LiH composites involved LiH suspension in THF under sonication and overnight stirring at 500 rpm, after which a THF solution of HAuCl_4_ was added and stirring continued for an additional 24 h, leading to the Au/LiH material after centrifugation and overnight drying by Schlenk line technique. Hydrogen absorption was carried out under 14.8 atm H_2_, while desorption was conducted under 0.2 atm pressure, utilizing Xe lamp illumination affording 100 °C local temperature [214].

Overcoming kinetic and thermodynamic barriers in the complex Li-N-H system (Equation (7)) led White et al., to study the Li_3_N effect on the LiNH_2_ + 2LiH composite behavior [215]. On this occasion, a kinetic analysis showed the rate-limiting step is the formation of H_2_ (g) at the surface of the core-shell structure Li_2_NH@Li_3_N [215]. Again, the use of TEM measurements was shown to be inappropriate for LiNH_2_ materials, due to decomposition upon prolonged electron beam exposure. The equilibria shown in Equation (7) already occur upon the exposure of Li_3_N to 10 bar H_2_ (200 °C, 2 h), but not at one bar H_2_, which only altered the α-to-β ration of Li_3_N [215].

(7) $${\mathrm{Li}}_{3}N+2H_{2}\overset{\Delta }{\Leftrightarrow }{\mathrm{Li}}_{2}\mathrm{NH}+\mathrm{LiH}+H_{2}\overset{\Delta }{\Leftrightarrow }{\mathrm{LiNH}}_{2}+2\mathrm{LiH}\overset{\Delta }{\Rightarrow }\frac{1}{2}{\mathrm{Li}}_{2}\mathrm{NH}+\frac{1}{2}{\mathrm{NH}}_{3}↑$$

Considering the gravimetric hydrogen densities required by DOE standards, LiH, MgH_2_ and AlH_3_ are the main binary systems proposed to date [216]. Silicon doping of LiH has shown a drastic reduction in decomposition temperature (ΔT = 230 K), and could store up to 5 wt.% H_2_ with release at 490 °C [216]. A nanostructured electrode of Co(OH)_2_ and silica was recently employed in Li-conductivity studies and showed the formation of active LiH species, although the material was not investigated for its hydrogen storage properties [217].

A series of Li-based materials was investigated by Xia et al., who grafted on graphene LiH by in situ reduction in nBuLi with H_2_ (110 °C, 50 atm), producing LiH@G. This nanocomposite LiH@G was further treated with B_2_H_6_ or AB/THF, and novel LiBH_4_@G and LiNH_2_BH_3_@G nanocomposites were thus obtained (Equation (8)) [114].

(8) $$G\overset{\mathrm{BuLi}}{\Rightarrow }\mathrm{LiH}@G\overset{B_{2}H_{6}}{\Rightarrow }{\mathrm{LiBH}}_{4}@G\;;\;G\overset{\mathrm{BuLi}}{\Rightarrow }\mathrm{LiH}@G\overset{{\mathrm{BH}}_{3}{\mathrm{NH}}_{3}}{\Rightarrow }{\mathrm{LiNH}}_{2}{\mathrm{BH}}_{3}@G\;$$

**Figure 4:**
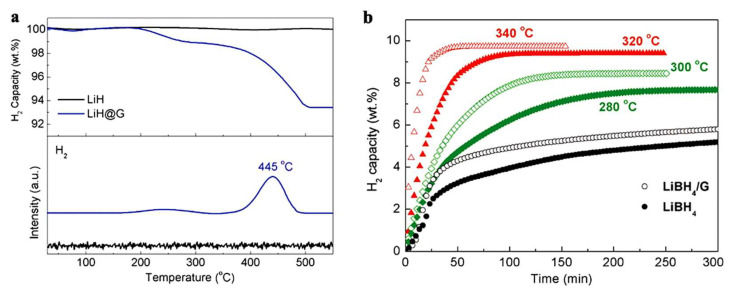
TG of as-prepared LiH@G (**a**), and dehydrogenation isotherm of LiBH_4_@G (**b**). Reprinted/adapted with permission from Ref. [114]. 2017, Wiley, under CC BY 4.0 license.

The 2D LiH nanosheets were about 2 nm thick and afforded a 6.8 wt.% H_2_ storage when loaded at 50 wt.% in the said graphene-based nanocomposite, which withstood structural integrity upon further hydride-to-borohydride transformation (Figure 4) [114].

Using HSAG (high surface area graphite) as scaffold, Wang et al., showed a 1.9 wt.% hydrogen storage at 200 °C for the composite LiH@HSAG, with reversible behavior at 300 °C and 60 bar H_2_ (Equation (9)) [133].

(9) $$\mathrm{HSAG}\overset{\mathrm{LiNp},{\;\mathrm{TiCl}}_{4}{\mathrm{THF}}_{2}}{\Rightarrow }\mathrm{LiH}@\mathrm{HSAG}\;$$

**Figure 5:**
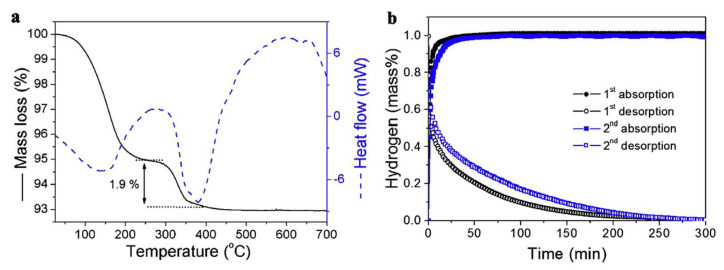
TGA/DSC for LiH@HSAG (**a**) and hydrogen desorption/absorption kinetics for LiH@HSAG at 350 °C (**b**). Reprinted/adapted with permission from Ref. [133]. 2016, Elsevier.

The morphology was tracked by SEM analysis and XRD diffraction, while hydrogenation data confirmed the modest 1.9 wt.% hydrogen storage by TGA (Figure 5).

This nanoconfinement approach in high surface area carbon (HSAG) of pore size 2–20 nm showed a high thermodynamic improvement, allowing for hydrogen release at 340 °C in LiH@HSAG rather than at the high 680 °C for pristine LiH [133].

### 6.2. MgH_2_

Due to its wide availability in nature, low cost, high gravimetric (7.6 wt.%) and volumetric (110 g/L) hydrogen storage capacities, the binary hydride, MgH_2_, is arguably the most studied metal hydride and Mg-based materials have been investigated exhaustively by a variety of research groups (Table 11) [34,43,47,53,55,57,61,65,77,78,80,92,94,95,98,102,103,109,110,115,118,121,122,124,130,134,137,146,147,149,158,168,169,170,172,177,196,198,201,202,204,205,208,209,210,211,212,214,216,218,219,220,221,222,223,224,225]. Synergistic effects of additives have been reviewed in the recent past: effect of nano-sized TMs (Ni, Cu, Fe, Co); salt addition in composites like MgH_2_ + 10 wt.% LaCl_3_; alloy formation Mg-La, Mg-Ni; incorporation in FeS_2_ nanospheres; dispersion effect of Nb_2_O_5_ catalysis; TiF_3_/TiO_2_/TiN/TiMn_2_ or Ti_3_C_2_ superior catalytic effects; Ni-based materials Mg@Ni binary nanocomposite; Mg_2_Ni alloy; Mg_2_NiH_4_; Ni@MgH_2_; NiB_2_/NiS/Ni_3_C/NiO/Ni_3_N/Ni_2_P; or carbon-based support influence (1D, 2D, 3D, graphene G, graphene oxide GO, MWCNT, etc.) (Table 11) [43,57,61,198,218,219,220,224]. The supporting role of a variety of carbonaceous hosts for MgH_2_ storage properties has been reviewed by Han et al., who underlined the added structural stability, catalytic effect and nanosizing structuring on metal hydrides, and magnesium hydride in particular [110]. Le et al., have recently viewed (2021) the nanoconfinement effects on H_2_-storage characteristics of MgH_2_ (and LiBH_4_) [47]. While conventional pressure-composition-temperature (PCT) data is time-consuming, an easier thermogravimetric analysis (TGA) was introduced by Zhou et al., to reliably determine abs./des. equilibrium temperature, and by using van’t Hoff equation to deduce reaction enthalpies and entropies: ΔH_abs_ = 79.8 kJ mol^−1^, ΔS_abs_ = 141.1 J mol^−1^K^−1^, and ΔH_des_ = 76.5 kJ mol^−1^, ΔS_des_ = 142.2 J mol^−1^K^−1^ for 5 at% VTiCr-catalyzed MgH_2_ [34]. Some research produced nano-assemblies MgH_2_@G (G = graphene) that was investigated as a material for high-performance Li-ion batteries (GMH composite with 50% MgH_2_ has reversible 946 mAhg^-1^ at 100 mAh^-1^ after 100 cycles) [103]. The necessity to better predict the behavior of Mg-containing clusters (Mg_n_H_m_) that emerge as the Mg/MgH_x_ (m < 2n) system matures, has led to a machine-learning (M-L) interatomic potential evaluation for Mg-H systems [223]. Wang and Huang have shown that the ML approach is able to accurately describe the diffusion coefficients and the Arrhenius type temperature dependence for 128 < t < 427 °C, a temperature range relevant for the Mg/MgH_2_ system in both pristine and nanoconfined conditions [223]. The diffusivity of H_2_ through Pd NPs deposited on Mg film has revealed that unlike the H_2_-impermeable MgO protective native film, the Pd-Mg interface can act as portals for hydrogenation of the Mg film [212].

**Figure 6:**
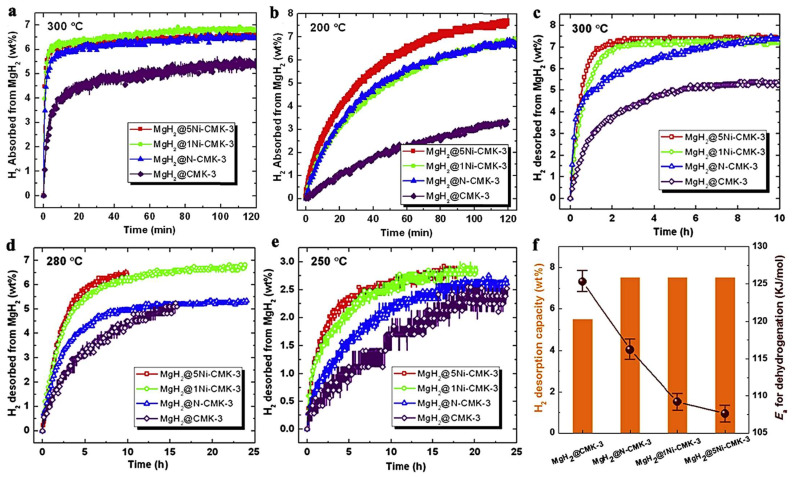
Hydrogenation kinetics of MgH_2_@CMK-3, MgH_2_@N-CMK-3 and MgH_2_@xNi-CMK-3 (x = 1 and 5) at 300 °C (**a**) and 200 °C (**b**) and under 19.74 atm. H_2_ backpressure. Hydrogen desorption profiles of the four investigated samples at 300 °C (**c**), 280 °C (**d**), 250 °C (vacuum, *p* < 0.01 atm) (**e**). Dehydrogenation of nanocomposites within two hours at 300 °C and corresponding desorption activation energies. E_a,des_ (**f**)_._ Reprinted/adapted with permission from Ref. [92]. 2017, Elsevier.

Jia et al., have utilized a Ni- or N-doped carbon scaffold for MgH_2_ nanoconfinement [92]. The carbonaceous support features high surface area, pore volume and narrow PSD (pore size distribution) and constitutes a C-replica of the mesoporous 2D-silica, SBA-15. Two Ni-loadings have been investigated: MgH_2_@xNi-CMK-3 (x = 1 and 5). Expectedly, the higher Ni-containing sample MgH_2_@5wt.%Ni-CMK-3 showed 7.5 wt.% storing capacity, whereas the MgH_2_@1wt.%Ni-CMK-3 and MgH_2_@ N-CMK-3 showed 6.5 wt.% hydrogen (Figure 6). The behavior of the nanocomposites has been investigated at 200, 250, 280 and 300 °C, and showed marked improvement scaling with temperature; at 300 °C, all three nanocomposites absorb 6 wt.% in 10 min (6.5 wt.% in 2h). The samples were degassed for 2 h at 350 °C prior to conducting absorption measurements (Figure 6) [92].

The enhancement in kinetics was obvious; van’t Hoff plot analysis revealed systematic decrease of the activation energy barrier in the order: MgH_2_@CMK-3 (125.3 ± 2.1 kJ mol^−1^) > MgH_2_@N-CMK-3 (116.2 ± 1.8 kJ mol^−1^) > MgH_2_@1Ni-CMK-3 (109.2 ± 1.3 kJ mol^−1^) > MgH_2_@5Ni-CMK-3 (107.6 ± 1.2 kJ mol^−1^) [92].

**Figure 7:**
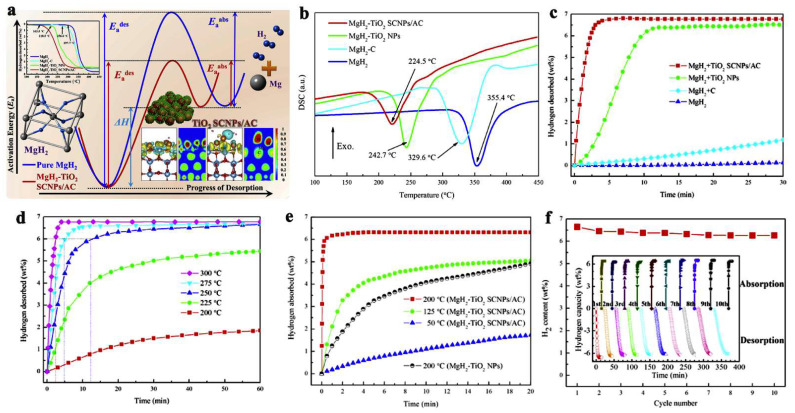
Schematic mechanistic approach in catalytic behavior of MgH_2_-TiO_2_ SCNPs/AC (**a**). DSC (**b**) of the investigated samples: ball-milled MgH_2_, MgH_2_-C, MgH_2_-TiO_2_ NPs and MgH_2_-TiO_2_ SCNPs/AC. Isothermal desorption curves of the four investigated samples at 300 °C (**c**); Isothermal desorption curves of MgH_2_-TiO_2_ SCNPs/AC and MgH_2_-TiO_2_ NPs at various temperatures in the range 50…300 °C (**d**,**e**); confirmation of reversible hydrogen storage capacity of MgH_2_-TiO_2_ SCNPs/AC at 300 °C recharging pressure of 50 bar H_2_ (**f**). Reprinted/adapted with permission from Ref. [95]. 2019, Elsevier.

Zhang et al., have dispersed TM-oxides (TiO_2_ in particular) on amorphous carbon to achieve excellent, reversible hydrogen storage capacity, releasing in 10 min. at 275 °C, 6.5 wt.% hydrogen (85.5% that of pristine MgH_2_) (Figure 7) [95]. Notably, the activation energies for desorption (E_a,des_) and absorption (E_a,abs_) have been considerably reduced compared to bulk magnesium hydride (Figure 7a). In a multi-fold enhancement strategy, the MgH_2_ was first dispersed on carbon (MgH_2_ + C), which showed modest improvements (<1 wt.% H_2_) over MgH_2_ bulk with no dehydrogenation in the same timespan (Figure 7c), TiO_2_ was used as additive for MgH_2_ to yield composites of MgH_2_ + TiO_2_ NPs, which surprisingly released ~6 wt.% H_2_ in 10 min [95]. Driven by these enhancements, nanocomposites of the type MgH_2_ + TiO_2_ SCNPs/AC were synthesized, which further improved hydrogen release/uptake: even at 50 °C, over the course of 20 min, ~1.5 wt.% H_2_ is released, whereas at 125 °C (~4.8 wt.%) and at 200 °C (6.5 wt.%) the kinetics is sped up considerably (Figure 7c–e). The rehydrogenation occurs within 5 min at 200 °C, and full recovery of the hydrogen storage capacity is achieved (6.5 wt.%). In addition, no appreciable hydrogen storage loss was recorded up to the 10th cycle (Figure 7f ) [95].

**Figure 8:**
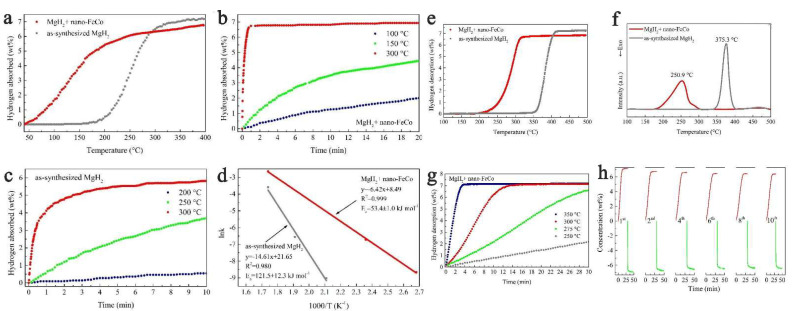
Non-isothermal hydrogenation curves (**a**); isothermal hydrogenation curves at different temperatures (**b**,**c**) and the corresponding Arrhenius plot of MgH_2_ with and without nano-FeCo (**d**); non-isothermal dehydrogenation curves (**e**); DSC curves with a heating rate of 5 °C min^−1^ of MgH_2_ with and without nano-FeCo (**f**); isothermal dehydrogenation curves of MgH_2_ + nano-FeCo composite at 250, 275, 300, 350 °C (**g**); dehydrogenation (in red) and rehydrogenation (in green) curves of MgH_2_ + nano-FeCo composite in the 1st, 2nd, 4th, 6th, 8th and 10th cycle (**h**). Reprinted/adapted with permission from Ref. [201]. 2019, Royal Society of Chemistry.

Using an FeCo nanocatalyst (mean size of 50 nm), Yang et al., synthesized composites MgH_2_ + nano-FeCo able to recharge to 6.7 wt.% hydrogen in one minute at 300 °C, and could desorb 6 wt.% (9.5 min, 300 °C) (Figure 8) [201]. In fact, even treatment under H_2_ backpressure at 150 °C produced 3.5 wt.% absorption in 10 min (Figure 8b). This highlights the importance of catalyst chosen, but also its morphology (nanosheets in the case of FeCo-nano). Plotting the Arrhenius equation also yielded the apparent activation energies: E_a,des_ = 65.3 ± 4.7 kJ mol^−1^ (60 kJ mol^−1^ reduction from pristine MgH_2_), and the absorption energy E_a,abs_ = 53.4 ± 1.0 kJ mol^−1^ (Figure 8d). Gratifyingly, the FeCo-catalyzed magnesium hydride composite was able to rehydrogenate fully and was tracked over the course of 10 hydrogen release/uptake cycles (Figure 8h) [201].

**Figure 9:**
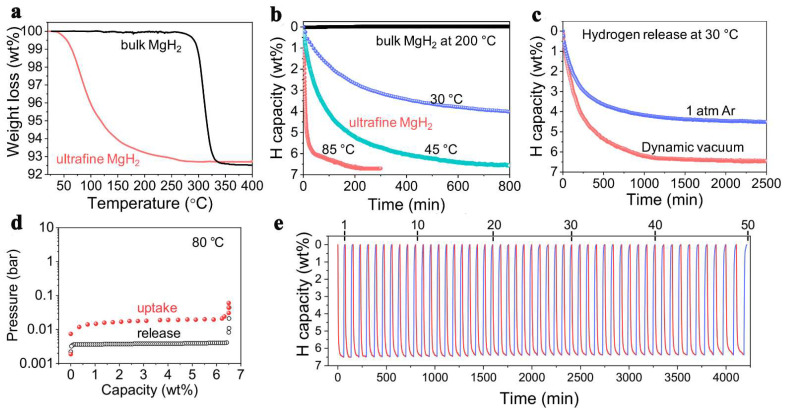
TGA (**a**), isothermal TGA dehydrogenation (**b**), isothermal TGA dehydrogenation under different conditions (**c**), PCI curves measured at 80 °C (**d**); cycling stability of non-confined ultrafine MgH_2_ (**e**). Reprinted/adapted with permission from Ref. [220]. 2018, Elsevier B.V.

The thermodynamic predictions that smaller size NPs will show the most important destabilization, Zhang et al., have produced ultrafine MgH_2_ that was able to release and recharge hydrogen under ambient temperature, with a very high hydrogen storage capacity of 6.7 wt.% (Figure 9) [222]. This capacity was checked over 50 cycles, and showed virtually the same high-capacity behavior (Figure 9). The conditions employed for reversible behavior were 360 min at rt (6.7 wt.%), or 60 min at 85 °C (6.7 wt.%), under 30 bar H_2_. This unexpectedly high storage capacity (65.6 g H_2_/L) surpasses even DOE’s requirement (50 gH_2_/L), and was possible solely on account of well-designed, size-restriction of MgH_2_ to nanoscale [222].

**Figure 10:**
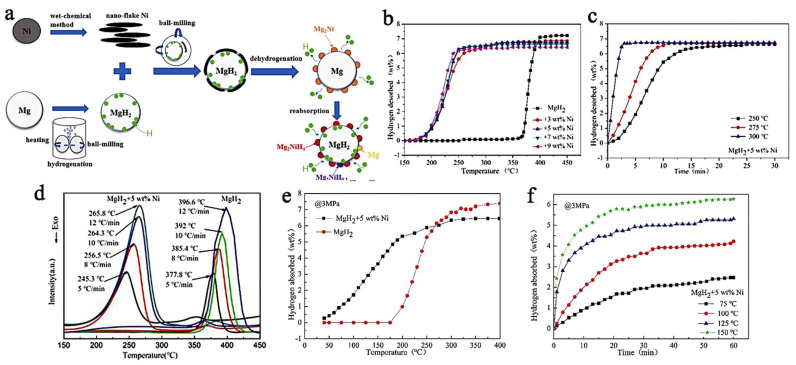
Preparation and reaction evolution in the MgH_2_+Ni composite (**a**); the rising temperature dehydrogenation curve (**b**); Isothermal dehydrogenation curves of MgH_2_ + 5 wt.% Ni at different temperatures (**c**); DSC curves of MgH_2_ + 5 wt.% Ni at different rates of increasing temperature (**d**); Non-isothermal hydrogenation curves of MgH_2_ with and without 5 wt.% Ni (**e**); Isothermal hydrogen absorption curves at different temperatures of MgH_2_ + 5 wt.% Ni (**f**). Reprinted/adapted with permission from Ref. [202]. 2021, Royal Society of Chemistry.

Using a nanoflake Ni catalyst, Yang et al., have synthesized MgH_2_ + 5 wt.% Ni, composites able to store 6.7 wt.% hydrogen (des., 300 °C, in 3 min) (Figure 10). The absorption was also very fast, achieving 4.6 wt.% at 125 °C in 20 min, under 29.6 atm H_2_ [202]. The results also translate into much lowered activation energies (Arrhenius plot): E_a,des_ = 71 kJ mol^−1^; E_a,abs_ = 28.03 kJ mol^−1^.

These results have been explained by means of the intermediate Mg_2_Ni intermediate, which is an intermetallic well-known in the Mg-Ni systems, and which absorbs rapidly H_2_ to form Mg_2_NiH_4_. This functions as an effective “hydrogen pump” (Figure 10a) (Equation (10)) [202].

(10) $${\mathrm{Mg}}_{2}\mathrm{Ni}+2H_{2}\overset{\Delta ,\;p}{\Leftrightarrow }{\;\mathrm{Mg}}_{2}{\mathrm{NiH}}_{4}\;$$

Decomposition of ^n^Bu_2_Mg typically used as an organometallic precursor to Mg/MgH_2_ NPs can follow two different steps, depending on the reaction temperature (Equations (11) and (12)).

(11) $${\left(C_{4}H_{9}\right)}_{2}\mathrm{Mg}\overset{160-265{}^{\circ}C}{\Rightarrow }\left(2-x\right)C_{4}H_{8\;\left(g\right)}+x\;{\left(C_{4}H_{8}\right)}_{\mathrm{surf}}{\mathrm{MgH}}_{2\left(s\right)}\;$$

(12) $${\left(C_{4}H_{8}\right)}_{\mathrm{surf}}{\mathrm{MgH}}_{2\left(s\right)}\;\overset{265-400{}^{\circ}C}{\Rightarrow }C_{4}H_{8\;\left(g\right)}+{\mathrm{MgH}}_{2\left(s\right)}\overset{\Delta }{\Rightarrow }{\;\mathrm{Mg}}_{\left(s\right)}+{\;H}_{2\left(s\right)}\;$$

**Figure 11:**
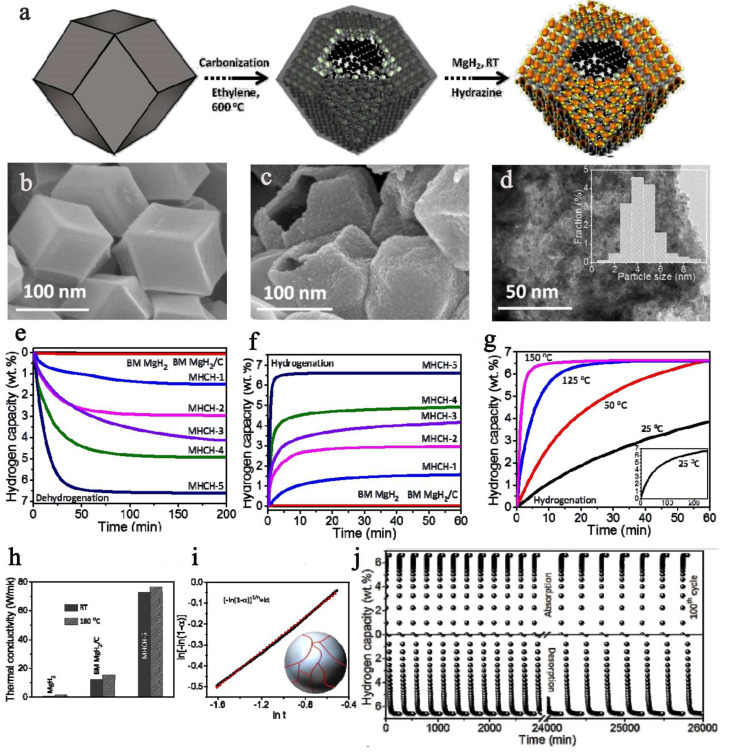
(**a**) Schematics displaying the self-assembled MgH_2_ on three-dimensional metal interacted carbon. (**b**) SEM image of prepared metal-interacted 3-D carbon; (**c**) SEM, (**d**) TEM images of the MHCH-5; (**e**) dehydrogenation of the as-synthesized MHCH samples at 180 °C in comparison to ball-milled MgH_2_ and MgH_2_/C; (**f**) Isothermal hydrogenation; (**g**) Hydrogen absorption of the MHCH-5 for different temperatures—the inset (**g**) shows the hydrogen absorption property of the MHCH-5 at 25 °C, over a long time period; hydrogenation and dehydrogenation were performed under hydrogen pressures of 10 bar and 0.01 bar, respectively; (**h**) Thermal conductivity variation in MHCH-5, MgH_2_, and ball-milled MgH_2_/C for ambient temperature and 180 °C; (**i**) The growth mechanism of MgH_2_ in MHCH samples correlating with a Johnson–Mehl–Avrami model. (**j**) Reversible hydrogen (under 10 bar H_2_ pressure) and dehydrogenation (under 0.01 bar H_2_ pressure) performance of the MHCH-5 at 180 °C. Reprinted/adapted with permission from Ref. [137]. 2017, The Royal Society of Chemistry; RSC Pub.

However small it might be, nanosized matter in general is also more reactive towards various gases and substrates, and Mg/MgH_2_ coupled system is no exception. Previous examples have overcome this downside by either pressing the nano-powders into pellets, or capping them with other reagents. There are however many reports where MgH_2_ has been introduced in the porosity of a carbonaceous host, such as the 3D activated carbon utilized by Shinde et al., to achieve a reversible hydrogen storage of 6.63 wt.% (Figure 11) [137]. Not only was the nanocomposite MgH_2_@3D-C storing hydrogen under relatively mild conditions 6.63 wt.% (five minutes, 180 °C), but the desorption was likewise fast (6.55 wt.%, 75 min, 180 °C), and perhaps more importantly, the nanoconfined MgH_2_ was air-stable thanks to the protective carbon shell [137]. To the observed enhanced kinetics and improved thermodynamic behavior contribute decisively the transition metal dispersed into the 3D carbon: NI>Co>Fe. Running in a continuous regime, the nanocomposite was able to cycle for about 435 h (more than 18 days), without a palpable decrease in the hydrogenation storage capacity (Figure 11) [137].

While typically reduction in ^n^Bu_2_Mg infiltrated into a nanoporous host to afford MgH_2_ NPs is carried out in heterogeneous conditions (under H_2_ pressure), Shinde used a mixed reductant system: TEA ((HOCH_2_CH_2_)_3_N)/NH_2_NH_2_ hydrazine to reduce Mg(II) to Mg(0) [137]. The synthetic procedure is nicely followed in Figure 11, and in this case, both scanning electron microscopy (SEM) and transmission electron microscopy (TEM) could be used for characterization, since the electron beam no longer hits directly the MgH_2_ NPs; thus, the risk of in-situ decomposition during data acquisition is minimized (Figure 11). The hydrogen storage capacity exceeds 6 wt.% in case of Ni-NPs deposited in the 3D-AC (MHCH-5), confirming the beneficial and synergistic role of Ni when used in conjunction with MgH_2_. The plausible intermediate Mg_2_Ni forms the coupled system Mg_2_Ni/Mg_2_NiH_4_ during hydrogenation, and this can be held responsible for the superior cycling behavior in case of MgH_2_@3D-AC (MHCH)-5(Ni), whereas this type of intermetallic is not common for Co or Fe [137].

The self-assembled MgH2 NPs are well embedded into the carbonaceous host, which plays a critical role in the overall performance of MHCH-5. It is implied, based on the thermal conductivity data (Figure 11h), that the carbon shell is important. The high thermal conductivity (70 W/mK), many times higher than that of MgH_2_ NPs themselves, induces a lower temperature gradient in the sample and a high heat transfer coefficient, thus contributing to the exemplary behavior of the sample during hydrogenation cycling [137].

### 6.3. AlH_3_

Alane (AlH_3_) is a metastable hydride, stabilized by the Al proneness to combine with oxygen and form a µm layer of Al_2_O_3_ ensuring chemical passivation. In bulk, AlH_3_ decomposes at 100–150 °C and the kinetics are reasonably fast, but the high H_2_ pressure required to achieve reversibility (10 GPa, 600 °C, 24 h; 10 GPa at 25 °C or 6 GPa at 300–380 °C by other accounts are all very high pressures) remains a hard obstacle to overcome (Table 12). Even so, mitigation of this drawback has been attempted by means of nanoconfinement [40,44,51,109,125,203,206,216,226,227,228,229,230,231,232,233]. Some results are pure theoretical results concerning the catalytic activity of nano-AlH_3_ [229] in the decomposition of 1,3,5,7-Tetranitro-1,3,5,7-tetrazocane, with simulated evolution of Al-clusters during the reaction [228], or decomposition of CH_3_NO_2_/nano-AlH_3_ composite [232].

The energetic bottleneck in hydrogenation of Al is the high activation barrier of H_2_ dissociation over the Al surface, therefore catalysts have been employed to lower this barrier by using TM dopants like Sc, V, Ti or Nb [229].

The reaction of LiH and AlCl_3_ was shown to be greatly sped up by using a 0.1 molar TiF_3_, when the final product obtained after five hours milling under Ar pressure was a nanocomposite of composition α-AlH_3_/LiCl-TiF_3_ [203]. Duan et al., have shown the critical role of TiF3 that acted as a seed crystal for α-AlH_3_. The pressure was also a crucial factor, as running the reaction under lower gas pressure only led to Al metal formation, without the envisioned hydridic phase (Equation (13)) [203].

(13) $$3\;\mathrm{LiH}+{\;\mathrm{AlCl}}_{3}\overset{\Delta }{\Rightarrow }\;3\;\mathrm{LiCl}+\mathrm{Al}+\frac{3}{2}H_{2}↑\;$$

**Figure 12:**
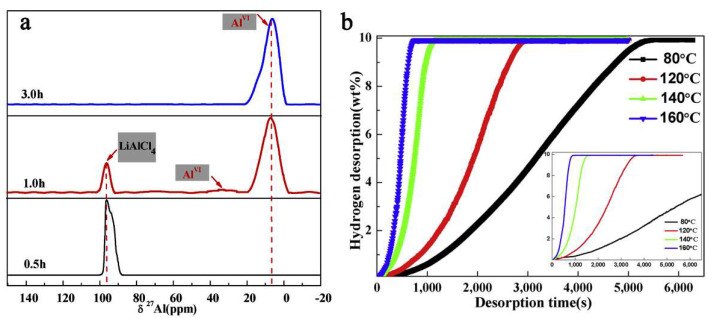
Solid state ^27^Al NMR for reactive mixture LiH/AlCl_3_/TiF_3_ (3:1:0.1) after ball milling for 0.5 h, one hour and three hours (**a**); hydrogen desorption curve for final composite α-AlH_3_/LiCl-TiF_3_ at temperatures 80 °C, 120 °C, 140 °C and 160 °C (inset shown for α-AlH_3_/LiCl without TiF_3_ addition) (**b**). Reprinted/adapted with permission from Ref. [203]. 2019, Elsevier B.V.

However, thermodynamic data showed a Gibbs free energy for the expected α-AlH_3_ formation of ΔG = −269 kJ mol^−1^, therefore thermodynamically possible at 298 K [203]. Furthermore, tracking the reaction by solid-state ^27^Al NMR spectra has shown the complex behavior of the reactive mixture (Figure 12) (Equation (14)).

(14) $$3\mathrm{LiH}+{\mathrm{AlCl}}_{3}\Rightarrow {\mathrm{LiAlCl}}_{4}+{\mathrm{AlH}}_{x}+2\mathrm{LiCl}\Rightarrow {\mathrm{LiAlCl}}_{4-x}H_{x}+2\mathrm{LiCl}\Rightarrow 3\mathrm{LiCl}+\alpha -{\mathrm{AlH}}_{3}$$

The kinetics are vastly improved, and raising the temperature above 120 °C allows for complete dehydrogenation in roughly 10 min (Figure 12).

**Figure 13:**
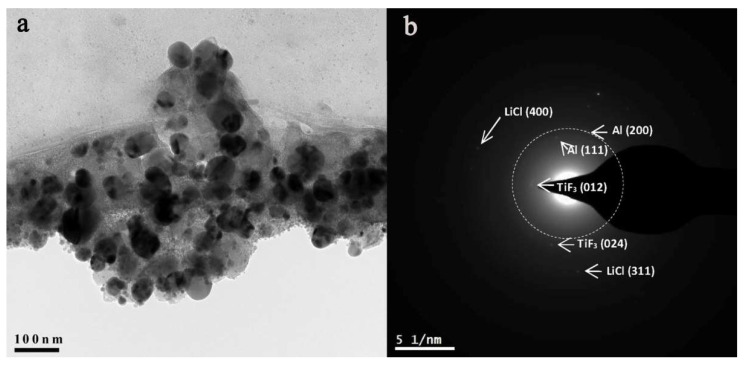
TEM image of α-AlH_3_/LiCl-TiF_3_ after dehydrogenation for 600 s at 160 °C (bright field TEM, (**a**); ED pattern, (**b**)). Reprinted/adapted with permission from Ref. [203]. 2019, Elsevier B.V.

After five hours of ball milling under Ar pressure and dehydrogenation at 160 °C for 600 s, the final composite (Figure 13) shows nanosized AlH_3_ (mean size of α-AlH_3_ was 45 nm, without traces of agglomerates).

The phase composition already shows formation of Al, consistent with the dehydrogenation reaction that had occurred. The report also highlighted the important role of the fluoride additive, as TiF_3_ reduced E_a_ of H-desorption to 52.1 kJ/mol [203].

**Figure 14:**
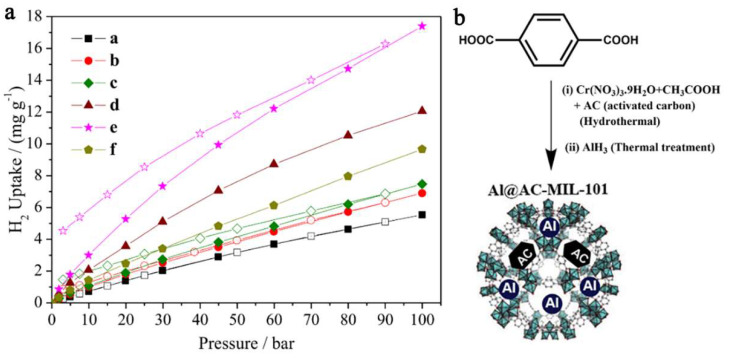
Hydrogen adsorption–desorption isotherms for (a) MIL-101; (b) AC-MIL-101; (c) AL@MIL-101; (d) Al@AC-MIL-101-A; (e) Al@AC-MIL-101-B; (f) Al@AC-MIL-101-C at 298K and pressures up to 100 bar H_2_ (closed symbols-Adsorption; open symbols: Desorption) (**a**). AlH_3_ introduction into MIL = 101 (**b**). Reprinted/adapted with permission from Ref. [40]. 2017, Elsevier Inc.

Nanoconfinement of alane in a Cr-based MOF (MIL-101) with Al-doping has led to a nanocomposite able to store and recharge at 298 K (ambient) and 100 bar H_2_, 17.4 mg H_2_/g (equivalent to 1.74 wt.% H_2_) [40]. The introduction of alane inside the MIL-101 pores was made via solvent infiltration from a THF solution of AlH_3_. In fact, the pristine MOF MIL-101 (3148 m^2^ g^−1^, 2.19 cm^3^g^−1^ and 2.5–3 nm pores) was shown to store 0.55 wt.% H_2_ under the same conditions. The hydrogen release profile from the investigated samples shows the improvement of nanoconfinement of AlH_3_ in MOF pores over the hydrogen release performance (Figure 14) [40].

The gravimetric storage capacity (17.4 mg H_2_ g^−1^ composite) was rather low considering DOE’s goals, due to the inability to increase Al-doping of the framework without crystallinity loss, and the role of AC additive became apparent in order to enhance hydrogen interaction with confined Al NPs [40].

**Figure 15:**
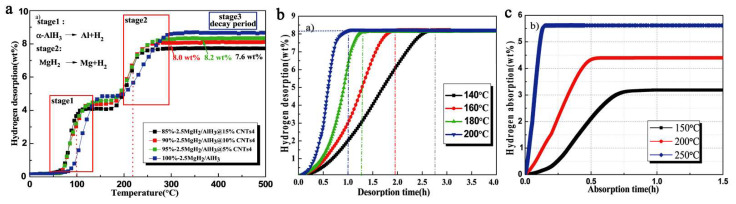
TPD (temperature programmed desorption) of 85%, 90% and 95%- 2.5MgH_2_/AlH_3_/CNTs4 and 100%-2.5MgH_2_/AlH_3_ (ball milling, BPR 20:1, 200 rpm, 1 h) (**a**); dehydrogenation isotherm of 95%-2.5MgH_2_/AlH_3_/CNTs4 under 10^−2^ Pa pressure custom vacuum system (**b**); Isothermal rehydrogenation curves of 95%-2.5MgH_2_/AlH_3_@CNTs4 at different temperatures under 5 MPa H_2_ pressure (**c**) [109]. Reprinted/adapted with permission from Ref. [109]. 2021, Royal Society of Chemistry.

In an attempt to improve upon previous results, Duan switched the nano-host to MWCNT (multi-walled carbon nanotubes) of high pore textural characteristics (550 m^2^ g^−1^, 6–8 nm diameter) and obtained by ball-milling xMgH_2_ + AlH_3_ (x = 1–4) nanocomposites MgH_2_/AlH_3_@CNT of crystal size 40–60 nm that released 8.2 wt.% H_2_ at 200 °C (60 min), and recharged to 5.61 wt.% H_2_ at 250 °C (10 min) (Figure 15) [109].

**Figure 16:**
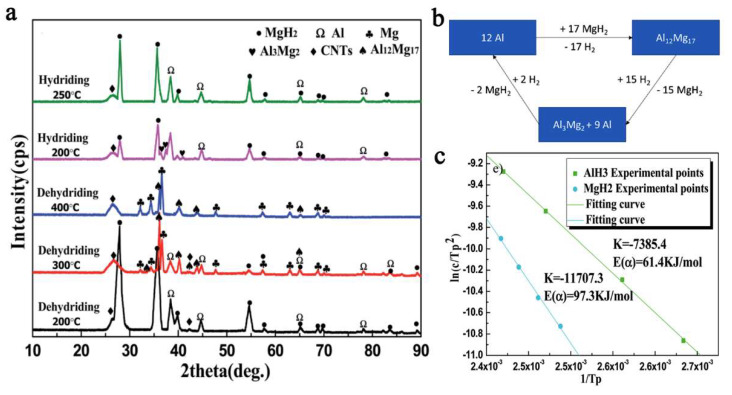
XRD pattern for 95%- 2.5MgH_2_/AlH_3_/CNTs4 after dehydrogenation at temperatures 200…400 °C (**a**); Al-tracking throughout the proposed mechanism, based on reaction data from ref. [109] (**b**) and Kissinger plot for deduction of E_a_ for hydrogenation of MgH_2_ and AlH_3_ (**c**) [109]. Reprinted/adapted with permission from Ref. [109]. 2021, Royal Society of Chemistry.

The Al metal produced in the first dehydrogenation stage of the composite (Figure 16) will react with MgH_2_ not yet dehydrogenated, to yield an intermetallic phase of Al_12_Mg_17_, which was confirmed by XRD data (Equation (15)).

(15) $$12\;\mathrm{Al}+17{\mathrm{MgH}}_{2}\Rightarrow {\mathrm{Al}}_{12}{\mathrm{Mg}}_{17}+17{\;H}_{2}↑$$

The reactions involved in the mechanistic proposal of the authors also allowed computation of the apparent activation energies (by Kissinger plot), which were of 97.3 kJ mol^−1^ for MgH_2_ and 61.4 kJ mol^−1^ for AlH_3_ (Figure 16c).

Wang et al., showed the potential of nanosizing by introducing (injection in HSAG of Et_2_O solution of freshly-made AlH_3_ from metathesis of LiAlH_4_ and AlCl_3_) [44]. Considering the 14 wt.% loading with AlH_3_ in the composite AlH_3_@HSAG (by ICP-OES), the expected hydrogen capacity was 1.4 wt.%. However, only 15% of the Al behaved reversibly and thus only an overall 0.25 wt.% storage could be attributed to the nanoconfined AlH_3_ [44]. Interestingly, during sample preparation, the composite was heated at 65 °C under Ar to yield α-AlH_3_ polymorph and minimize spontaneous decomposition of AlH_3_ [44]. Either way, the reduction in dehydrogenation onset to ~60° (60…270 °C with a peak at 165 °C) shows the effect of nanosizing, effectively reducing hydrogen release by 50 °C [44].

**Figure 17:**
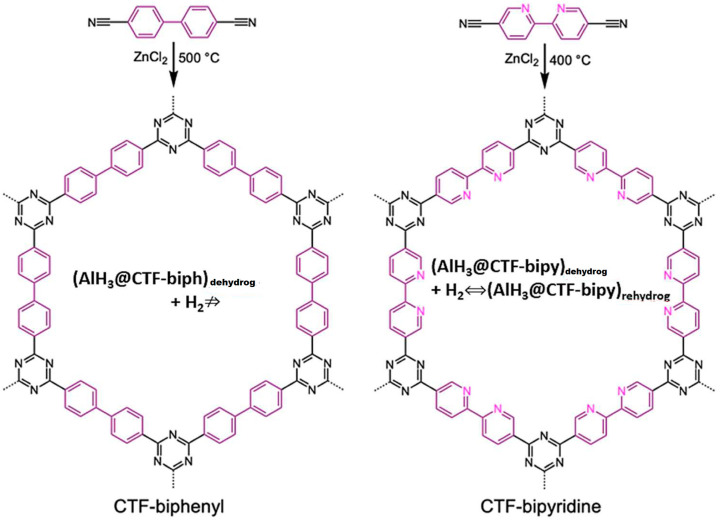
Construction of triazine-type CTF-biph and CTF-bipy used for alane nanoconfinement. Reversibility was only achieved for AlH_3_@CTF-bipy, presumably due to Al-complexation to N-atoms of bipyridyl moieties (shown inside the CTF frame). Reprinted/adapted with permission from [51].

Recently, using a triazine framework functionalized with bipyridine groups, CTF-bipy, a reversible behavior of alane in AlH_3_@CTF-bipy nanocomposite was observed at 700 bar H_2_ and 60 °C (although incomplete; Al signals still show in ^27^Al MAS-NMR) (Figure 17) [51].

**Figure 18:**
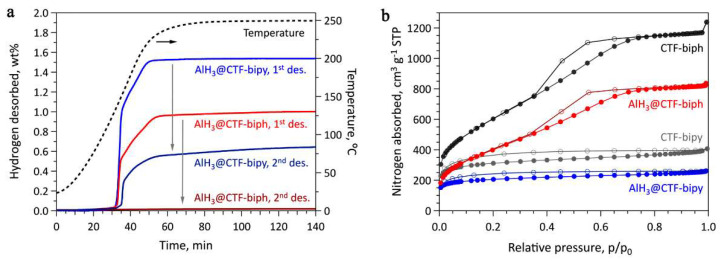
(**a**) Sievert data for CTF-based supported alane; (**b**) N_2_ sorption isotherms at 77 K for CTF-biph, AlH_3_@CTF-biph, CTF-bipy, and AlH_3_@CTF-bipy. Reprinted/adapted with permission from Ref. [51]. 2021, Wiley-VCH GmbH.

The EELS spectra of AlH_3_@CTF-biph and AlH_3_@CTF-bipy confirm that both contained aluminum, thus AlH_3_ introduction in the CTF-based frame was achieved. However, inherent oxidation had also occurred so the Al_2_O_3_ presence was also recorded by EELS data [51]. Although alane introduction into CTF-biph and CTF-bipy porosity was confirmed by N_2_ sorption isotherms (Figure 18), there was no reversibility in the case where CTF-biph was used as host [51].

Indeed, no reversibility was recorded in the absence of bipyridine groups (CTF-biph; biph = biphenyl), so the amino-functionality grafted on the covalent framework of the host was considered mandatory to achieve reversibility. This aspect was confirmed through DFT computations showing AlH_3_ or higher clusters—(AlH_3_)_2_, (AlH_3_)_3_, or (AlH_3_)_4_—coordinating the 2 N-atoms of the bipyridine group. However, the reversible H_2_ storage decreased from 1.44 wt.% (1st cycle) to 0.57 wt.% (4th cycle) [51].

**Figure 19:**
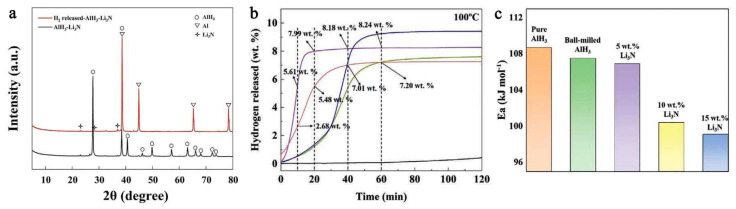
The XRD pattern (0.9AlH_3_-0.1Li_3_N)_dehydrog_ (**a**), the hydrogen release profile under isothermal conditions (100 °C) of (1 − x)AlH_3_-xLi_3_N (x = 0, 0.05, 0.1, 0.15) (**b**), and the calculated apparent activation energy (**c**). Reprinted/adapted with permission from Ref. [206]. 2022, Wiley-VCH GmbH.

Ball milling of a light metal nitride (Li_3_N) with AlH_3_ showed that a weakening of the Al-H bond is produced as a result of the milling process (a shift in XPS maximum), and that the hydrogen capacity decreases with the Li_3_N fraction: 9.04 wt.% (0.95AlH_3_-0.05Li_3_N), 8.71 wt.% (0.9AlH_3_-0.1Li_3_N) and 7.85 wt.% (0.85AlH_3_-0.15Li_3_N), compared to the ball milled pure AlH_3_ (9.86 wt.%) (Figure 19) [206].

Figure 19b shows the isothermal dehydrogenation of (1 − x)AlH_3_-xLi_3_N (x = 0.05, 0.1, 0.15) at 100 °C, confirming a decrease in H_2_ wt.% with the content of Li_3_N. The XRD pattern confirms that the sole dehydrogenation product of the composite is metallic Al (Figure 19a). The onset of dehydrogenation was conveniently reduced to 66.8 °C (0.95AlH_3_-0.05Li_3_N), thus approaching an operating regime suitable for FCEs. The beneficial role of lithium amide was confirmed by the apparent E_a_ which is strongly reduced (Figure 19c) [206].

### 6.4. TM-Hydrides

While main group metal hydrides are attractive due to metal abundance and low atomic weight of the metal (so higher wt.% H_2_ storage capacity), some TM (transition metals) have also been recently investigated by employing nanosizing effects (Table 13) [79,97,169,200,212,216,234]. The simplest and most classical model system to study TM-H interaction is the Pd-H system [200,234]. While the gravimetric storage capacity is too low for it to be considered for vehicular applications, the nature of Pd…H interaction has shed new light on thermodynamic predictions in Pd NPs forming PdH_x_, estimating cluster expansion, phase boundaries Pd/Pd…H, phase transitions (>400 K) and interfacial free energies by using DFT method [200,234]. Pd is often thought of as being able to absorb H_2_ like a sponge, reversibly absorbing more than 1000 times its own volume. In short, interaction of H_2_ with palladium comprises of H-H dissociation in atomic [H], diffusion of [H] into Pd_bulk_, where it occupies the free interstitial sites in *fcc* lattice of Pd, forming either an α-phase PdH_x_ (x < 0.03, rt) or the hydridic β-phase PdH_x_ (x > 0.03) [200]. The catalytic role of Ph-hydride has been recently harnessed in a complex Pd hydride CaPdH_2_, for semi-hydrogenation of C_n_H_2n−2_ (alkynes) to C_n_H_2n_ (alkenes) [79].

**Figure 20:**
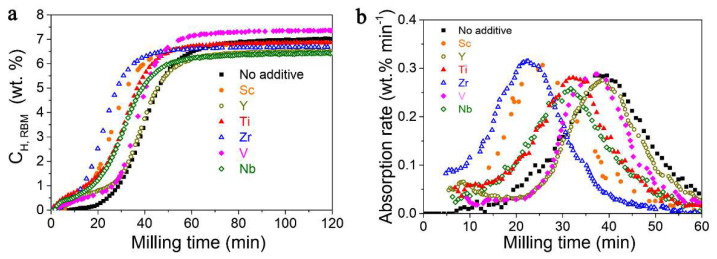
(**a**) Hydrogen uptake curves for 0.95 MgH_2_−0.05 (ETM)H_x_ during reactive ball milling; (**b**) absorption rate as derivative of hydrogen uptake curves. Reprinted/adapted with permission from Ref. [169]. 2019, Royal Society of Chemistry.

Rizo-Acosta et al., have addressed the issue of Mg/MgH_2_ slow kinetics by the addition of ETM (early transition metals: ETM = Sc, Y, Ti, Zr, V, Nb) to nanostructured MgH_2_ in a one-pot, mechanochemical reaction [169]. The influence of the milling time (0…120 min) over hydrogen wt.% storage capacity (Figure 20a) and absorption rate (Figure 20b) at 573 K has been studied and reveals that using 0.95 MgH_2_ –0.05 VH_2_, a 7.3 wt.% hydrogen uptake is registered, even higher than the experimental value for MgH_2_ (7.6 wt.% theoretical, 7.1 wt.% experimental) [169]. Moreover, the absorption rate is the fastest for 0.95 MgH_2_ –0.05 TiH_2_, with a shoulder in the sigmoidal shape due to (ETM)H_x_ formation, and varies in the order Y < V < Ti < Nb < Sc < Zr (Figure 20b). These hydrides (ScH_2_, YH_3_, TiH_2_, ZrH_2_, VH, NbH) are stable under experimental hydrogenation conditions and have a crystal size of ~10 nm, acting as effective catalysts for dehydrogenation (recombination of H atoms) and rehydrogenation (MgH_2_ nucleation due to MgH_2_/(ETM)H_x_ interface energies).

**Figure 21:**
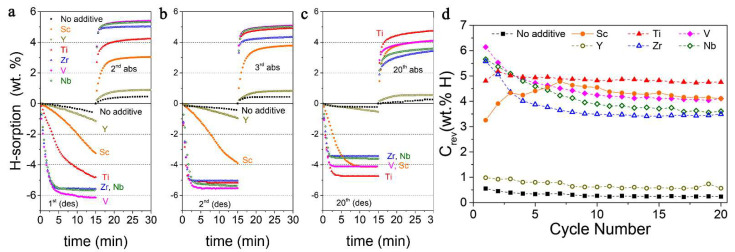
Hydrogen sorption curves recorded during the 2nd (**a**), 3rd (**b**) and 20th (**c**) absorption cycle for as-synthesized nanocomposites. Evolution of reversible hydrogen storing capacity with number of cycles (**d**). Reprinted/adapted with permission from Ref. [169]. 2019, Royal Society of Chemistry.

Notably, the reductive synthesis (300 °C, 7.89 atm H_2_) yields stabilization of the lower oxidation states of ETM, and mostly (ETM)H_2_ are produced, except for YH_3_ which affords the slowest desorption rate (0.06 wt.% min^−1^, 1 wt.% hydrogen release in 15 min under 0.296 atm H_2_). The best result was obtained for 0.95 MgH_2_−0.05 VH, when 6.1 wt.% (90% of the maximum) hydrogen was desorbed in 15 min (Figure 21) [169].

The most stable reversible capacity during cycling was achieved for 0.95 MgH_2_−0.05 TiH_2_ nanocomposite, which shows fast kinetics and does not fall below 4.8 wt.% even after 20 cycles (Figure 21). Additionally, no Mg-ETM-H ternary phases were observed [169].

A series of notable advances have been observed for complex hydrides as well, although their details are beyond the scope of this review. In short, metal tetrahydridoaluminates/alanates [14,51]: LiAlH_4_ [54,81,96,99,101,111,113,135,166,178], NaAlH_4_ [33,45,50,69,74,82,127,128,129,167,214,234,235], tetrahydridoborates/borohydrides [3,12,14,42]: LiBH_4_ [42,45,47,54,56,70,78,81,87,89,91,93,99,100,104,106,108,120,123,130,134,135,161,216,230,231,236,237,238,239,240], NaBH_4_ [49,74,216,236,241], Mg(BH_4_)_2_ [42,55,61,70,105,126,132,151,159,207,216,220,224,236,242,243], Ca(BH_4_)_2_ [116,216,236,243] and (TM)(BH_4_)_x_ [150,216], ammonia-borane NH_3_BH_3_ [36,38,63,64,75,85,86,88,90,139,140,141,142,143,144,153,154,156,162,171,175,197,213,244,245,246,247,248,249] and RCH reactive hydride composites [45,54,78,91,99,130,134,173,215,240,250] have been recently explored and improved thermodynamic and/or kinetic parameters have been reported [107,145,226,251,252,253].

## 7. Conclusions and Outlook

The urgency of a green, renewable and sustainable fuel to replace fossil fuels is more stringent today than ever. The metal hydrides constitute materials that possess intrinsically high gravimetric and volumetric hydrogen storage capacities, but their sluggish kinetics and poor thermodynamics still constitute an obstacle for the wide acceptance of their use in the fuel of the future. However, various strategies have been recently explored, and perhaps the most returns derive from basic shifts in thinking: oriented growth of MgH_2_ on catalytically active substrates; size-reduction in metal hydrides to few nm when thermodynamic destabilization works best; or usage of new class of catalysts of 2D-structure (MXenes)—they have all showed unexpectedly good results. There is clearly room for improvement in the fascinating field of metal hydrides, and research efforts ought to concentrate on improving nanoparticle system design, careful consideration of the incorporating matrix and selected hydrogenation/dehydrogenation catalysts, from both an economic and a feasibility point of view. Given the raw material scarcity but also reactivity and particular characteristics of some complex hydrides (like volatility of Al(BH_4_)_3_, or extreme toxicity of Be(BH_4_)_2_ etc.), the optimal hydrogen storage material will likely be based on magnesium nanoconfined in a carbonaceous host and/or catalyzed by Ti-based catalysts (such as TiO_2_, TiO, or MXenes). The realistic application of metal hydride systems is conditioned by a number of factors: (i) the discovery of a material that displays a reliably-reversible behavior in hydrogenation studies; (ii) consistent performance across hundreds of H_2_-absorption/desorption cycles; (iii) lower activation energies and consequently faster absorption/desorption kinetics and improved thermodynamics; (iv) consistently fast kinetics for fast refueling; (v) thermodynamic stability and material integrity to afford safe storage in a fuel tank; (vi) reasonable resistance to air and/or moisture; (vii) synthesis route moderately easy and preferably comprising of few steps; (viii) access to sufficient raw materials and limit amount of CRM (critical raw materials) used; (ix) reliable scaling-up of the lab demonstrator to a multi-KW tank capable to drive a vehicle for 500 km or more; (x) strong safety precautions and technological parameters implementation to afford a tank capable to store, release and withstand high H_2_ pressures (of more than 100 atm). Within this framework, the EU directives to limit CRM usage is expected to drive the research towards more-abundant metal sources such as Mg or Al (Mg was also included in the list of CRM from 2020, although currently it can be obtained in enough quantities). Noble metal catalysis (like Pd) will probably not become a commercial way of speeding up hydrogen delivery or the recharging of hydride-based fuels due to the associated cost. Other catalysts like MXenes can be produced on a larger scale, but the Ti-based material could also face soon shortages.

Nanoconfinement still offers general improvements across the board for hydride-based materials, but the choice of host is limited—among the classes of hosts presented in the current review, the most promising are carbonaceous frameworks and MOFs. Carbon-based materials can be tailored morphologically for hydride inclusion, and their cost is modest; however, this must be considered with care since a zero-carbon policy might imply soon that carbon should not be used as a host any longer. Even though it releases no CO_2_ in the atmosphere; there will be an associated cost with treatment of the end-of-life C-based fuel, and so the carbon footprint will not be negligible.

Considering these material, performance, safety and cost restrictions, the final choice for a viable, sustainable hydride-based material is a delicate one and only validation through a scaling-up proven in an operational environment could confirm whether it can be used on a large-scale tank for vehicular applications and afterwards adopted by industry. The ultimate goal is, without a doubt, to approach as much as possible the reversible, theoretical hydrogen capacity, and this is a joint venture of all the above considerations.

## Figures and Tables

**Table 1:** Recent progress in silica-based supports for nanoconfined hydrogen storage.

Silica Type	Hydrogen Storage Material	Nanoconfinement Method	Ref.
MSU-H	LiBH_4_	solvent infiltration	[16]
MCM-41	NaBH_4_	melt impregnation	[74]
MCM-41, SBA-15	LiBH_4_	melt impregnation	[87]
poly(acryalamide)-grafted mesoporous silica nanoparticles (PAM-MSN)	NH_3_BH_3_ (AB)	melt impregnation	[88]
SBA-15	Li_2_(BH_4_)(NH_2_).	melt impregnation	[89]
silica aerogel	NH_3_BH_3_ (AB)	aerogel drying and AB gas antisolvent precipitation	[90]
MCM-41, SBA-15	LiBH_4_-LiNH_2_	melt infiltration	[91]

**Table 2:** Recent progress in carbonaceous-based supports for nanoconfined hydrogen storage.

Carbon Type	Hydrogen Storage Material	Nanoconfinement Method	Ref.
MOFs incorporating activated carbon (AC) and aluminum doping	AlH_3_	solution impregnation method	[40]
Hollow carbon spheres (HCNs)	M(BH_4_)_x_ (M = Li, Na, Mg, Ca)	solvent impregnation (best results, lower T_d_), melt infiltration	[42]
Carbon aerogels with different porosities	Mg/MgH_2_	direct solvent-based synthesis of MgH_2_ from MgBu_2_	[53]
Core-shell CoNi@C	MgH_2_ obtained by hydriding combustion synthesis (HCS)	high energy ball milling under Ar atmosphere	[65]
Graphene	NaAlH_4_	solvent infiltration (THF; bottom-up strategy (90% loading)	[69]
Porous hollow carbon nanospheres	LiBH_4_-Mg(BH_4_)_2_ eutectic (LMBH)	melt-infiltration	[70]
xNi-CMK-3; N-CMK-3 (x = 1 and 5 wt.%)	MgH_2_	in situ generated from MgBu_2_ soln. in heptane	[92]
Double-Layered Carbon Nanobowl	LiBH_4_	melt infiltration	[93]
Carbon shell (2–3 nm thick)	Mg/MgH_2_	reactive gas evaporation	[94]
TiO_2_-decorated amorphous carbon (AC)	MgH_2_	ball milling	[95]
High Surface Area Graphite (HSAG)	LiAlH_4_	solvent infiltration/incipient wetness method	[96]
Porous carbon, High Surface Area Graphite (HSAG-500)	Mg_2_CoH_5_	bottom-up approach (Co^2+^ salt reduction, MgBu_2_ hydrogenation and solid-gas reaction Co + 2MgH_2_ + 0.5H_2_)	[97]
Graphene	MgH_2_	solvent-free, MgBu_2_ thermal decomposition	[98]
Resorcinol-formaldehyde carbon aerogel (RFC)	2LiBH_4_-LiAlH_4_	two-step melt-infiltration	[99]
Activated charcoal (AC)	LiBH_4_	melt-infiltration	[100]
NiCo_2_O_4_-anchored reduced graphene oxide (NiCo_2_O_4_@rGO)	LiAlH_4_	low-temperature solution method coupled with annealing treatment; to yield NiCo_2_O_4_@rGO nanocomposites	[101]
Nickel@nitrogen-doped carbon spheres (Ni@NCS)	MgH_2_	hydriding combustion and subsequent high-energy ball milling	[102]
Ultrathin, flexible Graphene (GR)	MgH_2_	bottom-up self-assembly strategy (from MgBu_2_ in C_6_H_12_)	[103]
Porous Hollow Carbon Nanospheres (PHCNSs)	LiBH_4_	mortar grounded, then melt infiltration (300 °C, 30 min, 100 bar H_2_)	[104]
Electrochemically synthesized reduced graphene oxide (erGO)	Mg-B	ball milling	[105]
Fe_3_O_4_@C, Multifunctional porous scaffold of carbon wrapped ultrafine Fe_3_O_4_	LiBH_4_	melting infiltration (300 °C, 30 min, 100 bar H_2_)	[106]
Activated carbon nanofibers (ACNF) impregnated with TiO_2_	LiBH_4_	mortar grinding (1:1, wt.), melt infiltration (310 °C at 5 °C/min rate under 60 bar H_2_, dwelling at 310 °C for 45 min, cooling to rt)	[108]
Carbon nanotube (CNT)	xMgH_2_/AlH_3_ (x = 1–4)	ball milling (200 rpm, 1 h, under H_2_ atmosphere) for xMgH_2_/AlH_3_; ball milling in steel container (1 h, under H_2_ atmosphere) for MgH_2_/AlH_3_@CNTs	[109]
Carbon nanoscaffolds (Graphite, CMK-3, Graphene, CNT)	MgH_2_	solvent, melt infiltration	[110]
N-doped CMK-3 carbon (NCMK-3)	LiAlH_4_	solution infiltration of LiAlH_4_ freshly recrystallized from diethyl ether	[111]
N-doped graphene hydrogels (resorcinol-formaldehyde)	LiBH_4_	ball milling (300 min, 400 rpm), melt impregnation (30 min, 300 °C, 60 bar H_2_)	[112]
N-Doped Graphene-Rich Aerogels Decorated with Ni and Co Nanoparticles	LiBH_4_	pre-mixing (mortar, pestle; 30 min), then melt impregnation (30 min, 300 °C, 60 bar H_2_).	[113]
Graphene sheets (G)	LiH (LiBH_4_, LiNH_2_BH_3_)	one-step solvothermal reaction of butyllithium supported by graphene in cyclohexane under a H_2_ pressure of 50 atm.	[114]
Graphene Nanosheet (G)	MgH_2_	solid-state reaction (metathesis MgCl_2_, LiH), ball milling (30 h, 0.5 MPa H_2_, 500 rpm)	[115]
Activated mesoporous carbon (MC-a)	Ca(BH_4_)_2_	incipient wetness method (0.1 M Ca(BH_4_)_2_.MTBE methyl tert-butyl ether, anhydrous)	[116]
Edge-Functionalized Graphene Nanoribbon (GNRs): unfunctionalized cGNR, nitrogen edge-doped N_2_-cGNR and N_4_-cGNR, and fluorenone GNR (f-cGNR)	Mg(/MgH_2_)	Rieke-like reaction (up to 98% Mg wt.%)	[117]
Ultrafine Ni nanoparticles dispersed on porous hollow carbon nanospheres (PHCNSs)	MgH_2 (_Mg_2_Ni/Mg_2_NiH_4_)	ball-milling (50 bar H_2_, 24 h, planetary ball mill QM-3SP4, Nanjing, 500 rpm, ball-to-sample weight ratio of 120:1)	[118]
Hydrogenated graphene (HG)	N/A	Li-reduction in graphene(G), then CH_3_OH hydrogenation	[119]
Graphene decorated with Ni nanocrystals	LiBH_4_	solvothermal reaction (50 bar H_2_ at 100 °C, 24 h, continuous stirring); ^n^BuLi hydrogenation (to LiH) and C_6_H_15_NBH_3_ reaction (to LiBH_4_-C_6_H_15_N); Cp_2_Ni (for Ni)	[120]
Defected graphene oxide (GO) or reduced graphene oxide (rGO)	Mg/MgH_2_	in situ generation of Mg from a THF soln. of Cp_2_Mg	[121]
Reduced graphene oxide (rGO)/Li foil	Mg/MgH_2_	direct solvent-based synthesis of MgH_2_ from MgCp_2_	[122]
Carbon Matrix	LiBH_4_	melt-impregnation	[123]
1D Carbon Matrix (Fishbone Shaped): CNF, GNF	Mg/MgH_2_	direct solvent-based synthesis of MgH_2_ from sonicated, solvent(THF)-impregnated MgCp_2_-CNF/GNF	[124]
Nickel-Containing Porous Carbon Sheets (Ni-PCSs)	LiAlH_4_, NaAlH_4_, and Mg(AlH_4_)_2_	pre-mixing in mortar (15 min.), high energy ball-milling (SPEX M8000 mixer/mill, 15 min.) w/ball-to-powder weight ratio 40:1.	[125]
Reduced graphene oxide (rGO)	Mg(BH_4_)_2_	in situ generation of rGO/Mg(BH_4_)_2_: rGO slurry with 1 M MgBu_2_ in heptane, added over BH_3_·S(CH_3_)_2_.	[126]
MWCNT (w/TiO_2_ 2 mol% relative to NaAlH_4_)	NaAlH_4_	physical mixture; PEIS/MWCNT/NaAlH_4_; polyaniline (Pani) or sulfonated polyetherimide (PEIS) as polymer matrices	[127]
Nitrogen-Doped Nanoporous Carbon Frameworks (N-doped NPC)	NaAlH_4_	pre-mixing (mortar/pestle, 10 min), melt infiltration (Sievert apparatus, 190 bar H_2_, 45 min, 200 °C)	[128]
Graphene oxide (GO) framework	NaAlH_4_	incipient wetness impregnation	[129]
Activated carbon (AC)	2LiBH_4_-MgH_2_	milling 2LiBH_4_:Mg in stainless-steel vial planetary ball mill; 20:1 ball-to-powder weight ratio (BPR), 10 h milling time, 580 rpm	[130]
Ordered mesoporous carbon structures (CMK)	N/A (Ni NP)	Ni NPs inserting by wetting the CMK structures	[131]
ultrafine Ni nanoparticles in a mesoporous carbon matrix (MC-N_iinsitu_)	Mg(BH_4_)_2_	Mg(BH_4_)_2_ (45 wt.%) solution (THF, Et2O) slowly impregnated into the MC variant	[132]
High surface area graphite (HSAG)	LiH	catalytic hydrogenation of lithium naphthalenide (for LiH), stirring at 400 rpm, 0.35 MPa H_2_, 40 °C, aged overnight.	[133]
Fe-benzenetricarboxylate (Fe-BTC)	NaAlH_4_	solution infiltration using tetrahydrofuran (THF)	[134]
Activated carbon nanofibers (ACNF)	LiBH_4_-LiAlH_4_	solution impregnation of LiAlH_4_ (Et_2_O) then melt infiltration of LiBH_4_ (310 °C, 110 bar H_2_, 45 min.)	[135]
Carbon aerogel (CA) by resorcinol (R) and formaldehyde (F) process	N/A	triethylamine (as catalyst)	[136]
3-D activated carbon (M-3D C)	MgH_2_	solvent-reduction (NH_2_NH_2_) of a slurry MgBu_2_ (1 M, heptane) in M-3D C	[137]
Reduced graphene oxide (rGO)/metal nanocrystal multilaminates	Mg/MgH_2_	solution-based co-reduction method of MgCp_2_/GO with lithium naphthalenide solution (2 h stirring, then 20 min centrifuged @10,000 rpm)	[138]
ZIF-67-Derived Co@Porous Carbon	NH_3_BH_3_ (AB, Ammonia Borane)	infiltration	[139]
Carbon nanotube arrays (CMK-5)	AlH_3_ and NH_3_BH_3_	pre-mixed (mortar, hand-milling); solvent (THF) infiltration into CMK-5.	[140]
carbon nanomaterials MDC (based on calcined MOF-5)	NH_3_BH_3_	solvent infiltration	[141]
Ice templating sheets of graphene oxide (GO) or partially reduced graphene oxide (rGO)	NH_3_BH_3_	solvent infiltration (AB infiltrated to a solvent suspension of GO)	[142]
Bio-derived micro/mesoporous carbon with well-organized pores (TiO_2_/B co-catalysts)	NH_3_BH_3_	solvent immersion (AB methanol solution into C-TiO_2_(B)), then vaporization	[143]
Microporous carbon (ECMC, narrow PSD, obtained by CVD from ethylene-filled Zeolite EMC-2)	NH_3_BH_3_	solvent infiltration (of AB methanol solution to ECMC)	[144]
V_2_O_3_-supported cubic C-nanoboxes	MgH_2_	ball milling (500 rpm, 24 h, BPR:120:1, 50 bar H_2_).	[146]

**Table 3:** Recent progress in MOF-based nanoconfined hydrogen storage systems.

MOF Type	Hydrogen Storage Material	Nanoconfinement Method	Ref.
Cu-BDC(DMF) (BDC = benzenedicarboxylate; DMF-dimethylformamide, used as removal/capping solvent)	AB (NH_3_BH_3_)	hand grinding (5 min, under Ar); AB: Cu-BDC(DMF) weight ration: 1:20, based on pore filling estimation	[39]
MIL-101-NORIT-RB3 decorated (an activated carbon AC added in situ during synthesis of MOF)	AlH_3_	solvent impregnation (THF, under Ar)	[40]
Various MOFs (of type MOF-5, MIL, UiO, ZIF, IRMOF etc.)	Pg/PdH_2_	Various: Liquid impregnation, Metal-Organic Chemical Vapour Deposition; Sol-Gel; Double Solvent Method	[68]
HKUST-1, IRMOF-1, IRMOF-10, UiO-66, UiO-67, and MIL-53(Al), MIL-101, MOF-74(Mg)	AB (NH_3_BH_3_), NaAlH_4_, MH_x_ (M = Li, Na, Mg, Ca, Al)	solvent- and melt infiltration	[86]
Nb_2_O_5_@MOF (Zn-based MOF, ZIF-8 (Zn(2-methylimidazole)_2_))	MgH_2_	ball milling (400 rpm, 4 h, ball to powder ratio 40:1) yielding MgH_2_@7 wt.% Nb_2_O_5_@MOF	[147]
MOF-5, MOF-177, HKUST-1, NOTT-100, Mg-IRMOF-74-I, NiIRMOF-74-I, Mg-IRMOF-74-II and Ni_2_(mdobdc)	Mg/MgH_2_; Ni/NiH_2_	Hydrogen release/uptake in Ni-based MOFs	[148]
Ni-MOF scaffold (Ni_2_(TMA), TMA-trimasic acid)	MgH_2_	in-situ synthesis; infiltration of MgBu_2_ (1 M in heptane) in Ni-MOF porosity, hydrogenation (453 K, 4.8 MPa H_2_, 20 h) to yield MgH_2_@Ni-MOF	[149]
UiO-66 (Zr_6_O_4_(BDC)_6_, BDC = 1,4-benzenedicarboxylate)	Ti(BH_4_)_3_	gas adsorption of Ti(BH_4_)_3_ at dry-ice conditions (N_2_-carrier gas) into UiO-66	[150]
UiO-67bpy (Zr_6_O_4_(OH)_4_(bpydc)_6_ with bpydc^2–^ = 2,2′-bipyridine-5,5′-dicarboxylate)	Mg(BH_4_)_2_	solvent impregnation	[151]
Various (High-throughput molecular simulations)	N/A	theoretical study (machine learning)	[152]
IRMOF-1, IRMOF-10, UiO-66, UiO-67, and MIL-53(Al)	AB (NH_3_BH_3_)	solvent infiltration (CH_3_OH)	[153]
MIL-53	AB (NH_3_BH_3_)	incipient wetness impregnation method (CH_3_OH saturated solution)	[154]
MIL-101-NH_2_ (Al)	Al/AlH_3_	solvothermal treatment involving N,N-dimethylformamide (DMF) as solvent	[155]
MOF-5	M/MH_x_	post-confinement, in-situ confinement, double-solvent method (better efficiency)	[156]
MOF = ZIF-8, ZIF-67, MOF-74	Mg/MgH_2_	in situ reduction in Mg^2+^-decorated MOFs by NpLi solution in THF	[157]

**Table 4:** Recent progress in oxides, sulfides and nitrides-based hosts for nanoconfined hydrogen storage systems.

Metal Oxide/Sulfide/Nitride	Hydrogen Storage Material	Nanoconfinement Method	Ref.
CoS nano-boxes (ZIF-67-derived)	MgH_2_	infiltration MgBu_2_ (1 M in heptane; 1000 rpm, 48 h), followed by hydrogenation (453 K, 4.8 MPa H_2_, 24 h)	[80]
Al-SBA-15, γ -Al_2_O_3_	LiBH_4_-LiNH_2_	melt infiltration	[91]
Metal oxide nanoparticles (TiO_2_) anchored on amorphous carbon (SCNPs/AC)	MgH_2_	in-situ pyrolysis assisted with quickly cooling	[95]
NiCo_2_O_4_-anchored reduced graphene oxide (rGO)	LiAlH_4_	low-temperature solution method coupled with annealing treatment; to yield NiCo_2_O_4_@rGO nanocomposites	[101]
Fe_3_O_4_@C, Multifunctional porous scaffold of carbon wrapped ultrafine Fe_3_O_4_	LiBH_4_	melting infiltration (300 °C, 30 min, 100 bar H_2_)	[106]
Nb_2_O_5_@MOF (Zn-based MOF, ZIF-8 (Zn(2-methylimidazole)_2_))	MgH_2_	ball milling (400 rpm, 4 h, ball to powder ratio BPR 40:1) yielding MgH_2_@7 wt.% Nb_2_O_5_@MOF	[147]
Ni/CoMoO_4_ nanorods	MgH_2_	ball milling (400 rpm, BPR: 60:1, 6 h); MgH_2_ is the *host* for NiCoO_4_/NiMoO_4_ nanorods to yield MgH_2_-10 wt.% Ni/CoMoO_4_	[158]
Al_2_O_3_	γ-Mg(BH_4_)_2_	Atomic Layer Deposition (ALD)	[159]
B_2_O (Metal-Decorated Honeycomb Borophene Oxide)	Li/LiH; Na/NaH and K/KH.	Theoretical study: dispersion corrected density functional theory (DFT-D2)	[160]
Al_2_O_3_	LiBH_4_-LiI	melt infiltration (50 bar H_2_, 295 °C, 3 °C min^−1^, 30 min); 4LiBH_4_:LiI–manual grinding in mortar, added to Al_2_O_3_ (130% pore filling)	[161]
(3D) boron nitride (BN)	AB (NH_3_BH_3_)	solvent impregnation of AB (6.92 M in THF) into mBN1000 and mBN1450	[162]
TiO_2_ (anatase)	MgH_2_	crystal-facet-dependent catalysis ({001} and {101})	[172]

**Table 5:** Recent progress in oxides, sulfides and nitrides-based hosts for nanoconfined hydrogen storage systems.

Metal as Host or Component	Hydrogen Storage Material	Nanoconfinement Method/Obs.	Ref.
Al	Al/AlH_3_@MIL-101-NORIT-RB3 decorated	solvent impregnation	[40]
Pd	Pd@MOF	Various: Liquid impregnation, Metal-Organic Chemical Vapour Deposition; Sol-Gel; Double Solvent Method	[68]
TiC_x_/Mg	Mg/MgH_2_	reactive gas evaporation	[94]
N-doped graphene	LiBH_4_	ball milling	[112]
Mg nanocrystals	Mg/MgH_2_@GO; Mg/MgH_2_@rGO	LiNp reduction in Cp_2_Mg/(r)GO slurry in THF. Various degrees of GO reduction (to rGO) to fine tune H_2_ storage properties by morphology modification of Mg confined in xGO/(1-x)rGO matrix.	[121]
Mg	Mg/rGO	One-step growth of Mg particles; chemical reduction in Cp_2_Mg by Li-methyl-naphtalenide (LiNp^Me^) in THF, followed by addition of the reactive mixture over single layer GO (30 min sonication). Mg w/high-index {21̅1̅6} crystal surface exhibits increased hydrogen absorption up to 6.2 wt %.	[122]
Mg	GO/Mg/MgH multilaminates	solution-based co-reduction method of MgCp_2_/GO with NpLi	[138]
Mg@(rGO/Ni)	Mg/MgH_2_	in situ reduction in (Cp_2_Mg and Cp_2_Ni)@GO,THF-sonicated slurry, with a THF sol. of LiN; 6.5 H_2_ wt.% of total composite; H_2_ uptake under 1 bar H_2_.	[163]
Pd	Pd-Based Alloy Nanoparticles *RhPd-H NPs); PdH_0.43_ NPs (when np Pd used, control experiment)	one-pot solvothermal method-reduction of acetylacetonates Pd(acac)_2_ and Rh(acac)_3_ in mixed benzyl alcohol /acetaldehyde solvents with polyvinylpyrrolidone (PVP), at 180 °C in 30 min. RhPd confirmed by EDX. (111) diffraction peak outside that of either Rh/Pd, implying an expanded structure due to interstitial H atoms.	[164]
Mg (as matrix)	Mg/MgH_2_	(review) of solid-state processing: physical vapor deposition, powder blending and consolidation, and additive manufacturing.	[165]
Raney Ni (3 nm pore size) as host	NaAlH_4_ to form NaAlH_4_/Raney Ni	wet impregnation	[166]
Al/Ti (Ti-based doped porous Al scaffold)	NaAlH_4_/Al	melt-infiltrated	[167]
Co	2MgH_2_-Co (Mg_2_CoH_5_ and Mg_6_Co_2_H_11_)	compression to pellets (4.43 wt.% hydrogen storage) vs. powder (2.32 wt.% capacity)	[168]
Mg	MgH_2_ and ETM hydrides (ScH_2_, YH_3_, TiH_2_, ZrH_2_, VH and NbH)	mechanochemistry under hydrogen gas; 5 mol% of Early Transition Metals (ETM = Sc, Y, Ti, Zr, V, and Nb) as hydrogenation catalysts	[169]
Mg–Ti	Mg–Ti–H nanoparticle.(MgH_2_ andTiH_2_ crystalline phases)	gas-phase condensation of Mg and Ti vapors under He/H_2_ atmosphere	[170]
Ni	AB/Ni matrix	NiCl_2_ reduction to Ni(0) on the surface of AB nanoparticles (1–7 nm)	[171]

**Table 6:** Examples of gas-selective H_2_-permeable polymers used as covering shells for hydrogen storage systems.

H_2_-Permeable Polymers	Hydrogen Storage Material	Nanoconfinement Details	Ref.
poly(acrylamide) (PAM)-grafted mesoporous silica nanoparticles (MSNs)	ammonia borane (AB)	solution infiltration (stirring of THF solution of AB and polymer for 2 h), to produce AB-PAM-COOH-MSNs and AB-PAM-COOHMSNs	[88]
polyaniline (Pani) or sulfonated polyetherimide (PEIS) as polymer matrices	NaAlH_4_	PEIS/NaAlH_4_ (70/30 wt.%): solution infiltration of NaAlH_4_ added over dispersed MWCNTs in NMP-solubilized PEIS (30 min, 40 °C). Pani/NaAlH_4_: dispersion of components (50 wt.%), w/2 mol.% TiO_2_ as catalyst	[127]
mesoporous polystyrene	various metal hosts	post-confinement strategy	[156]
Adaptive TPX™ Polymer Scaffold	Li-RHC (2LiH + MgB_2_ + 7.5(3TiCl_3_·AlCl_3_))	ball milling of 2LiH + MgB_2_ + 7.5 (3TiCl_3_·AlCl_3_) and a solution of TPX^TM^ in cyclohexane	[173]
PTFE polytetrafluoroethylene; PMMA poly(methyl-methacrylate)	Pd or Pd_70_Au_30_ alloy	Pd@PTFe, Pd_70_Au_30_@PTFe, (Pd@PTFE@PMMA) acting as (tandem) sensors	[174]
short-chain polyethylene oxide (PEO or PEG)	AB (NH_3_BH_3_)	slow interaction of AB and PEO powders (microscope slide, 10 months. rt) forms ammonia borane–polyethylene oxide cocrystal (5 PEO monomers per AB molecule)	[175]

**Table 7:** Examples of MXenes used as hosts for hydrogen storage systems.

MXene Type	Hydrogen Storage Material	Nanoconfinement Method	Ref.
TiC_X_	Mg/MgH_2_	reactive gas evaporation method	[94]
Ti_3_C_2_T_x_ (T = surface termination: OH, O or F)	Ni@C spheres	in situ confinement strategy	[156]
Ti_3_C_2_T_x_ (T = F)	N/A	hydrogen trapping (physisorption, chemisorption, and Kubas type particle interaction)	[176]
Multilayer Ti_3_C_2_ (ML-Ti_3_C_2_)	MgH_2_	ball milling MgH_2_ + ML − Ti_3_C_2_	[177]
Ti_3_C_2_	LiAlH_4_	ball milling LiBH_4_-Ti_3_C_2_ (1 wt.%, 3 wt.%, 5 wt.%, 10 wt.%, and 15 wt.%); (planetary ball mill Retsch PM 400, under Ar, 250 rpm, 10 h, BPR 250:1); doping strategy to LiAlH_4_, yielding LiAlH_4_ + 5 wt.% Ti_3_C_2_	[178]
Ti_3_C_2_	4MgH_2_-LiAlH_4_	mechanical milling 4MgH_2_-LiAlH_4_ with additive Ti_3_C_2_ (10 wt.%) in planetary ball mill (24 h, 450 rpm, BPR 40:1, under Ar), forming 4MgH_2_-LiAlH_4_-Ti_3_C_2_ nanocomposites	[179]
Ti_3_C_2_	LiH + MgB_2_	ball milling	[180]
Nb_4_C_3_T_x_	MgH_2_	ball milling MgH_2_-5 wt.%Nb_4_C_3_T_x_; chemical exfoliation of Nb_4_C_3_T_x_	[181]
Cr_2_C	N/A	First-principles studies (7.6 wt.% H_2_)	[182]
Ti_3_C_2_	NaAlH_4_	NaAlH_4_-7 wt.% Ti_3_C_2_	[183]
(Ti_0.5_V_0.5_)_3_C_2_	MgH_2_	MgH_2_-10 wt.% (Ti_0.5_V_0.5_)_3_C_2_	[184]
Ti_3_C_2_	LiBH_4_	40% Ti_3_C_2_ composite	[185]
Ti_3_C_2_	NaH/Al (Ti-doped NaAlH_4_)	NaH/Al–Ti_3_C_2_	[186]
Ti_3_C_2_	Mg(BH_4_)_2_	Mg(BH_4_)_2_-40 wt.% Ti_3_C_2_ composite	[187]
C@TiO_2_/Ti_3_C_2_	NaAlH_4_	annealing Ti_3_C_2_ MXene under C_2_H_2_ atmosphere; 10 wt.% C@TiO_2_/Ti_3_C_2_ catalyzing NaAlH_4_	[188]
Ti_3_C_2_	Mg(BH_4_)_2_	ball-milling method; Mg(BH_4_)_2_–40Ti_3_C_2_	[189]
NbTiC solid-solution MXene	MgH_2_	MgH_2_-9 wt.% NbTiC	[190]
Ti_3_C_2_	2LiH + MgB_2_/2LiBH_4_ + MgH_2_ (RHC-system)	ball milling	[191]
Ti_3_C_2_/TiO_2_(A)-C	MgH_2_	ball milling; sandwich-like Ti_3_C_2_/TiO_2_(A)-C prepared by gas–solid method	[192]
Ti_3_C_2_	Mg/MgH_2_	ball milling (50 bar H_2_, 24 h) producing MgH_2_-*x* wt.% Ti_3_C_2_ nanocomposites (*x* = 0, 1, 3, 5 and 7)	[193]
Ti_2_N	N/A	first-principles calculations; 2.656–3.422 wt.% hydrogen storage capacity, ambient conditions	[194]

**Table 8:** Examples of host decoration/doping and hydride substitution in nanosized systems.

Host Doping/Hydride Substitution	Hydrogen Storage Material	Nanoconfinement Method	Ref.
Alkali/Alkaline Earth Metals (AM)	hydrides of lightweight elements (HLEs)	development of AM amide-hydride composites	[19]
Pd	Pd/PdH_x_@MOF	complex interaction Pd…H	[68]
metal (Ni) or non-metal (N)-doping of carbon scaffold	MgH_2_	xNi-CMK-3; N-CMK-3 (x = 1 and 5 wt.%)	[92]
Ni@N-doped carbon spheres	MgH_2_	hydriding combustion and subsequent high-energy ball milling	[102]
Nitrogen-Doped Carbon Host	LiAlH_4_	solution infiltration	[111]
N-doped graphene in resorcinol-formaldehyde	LiBH_4_	ball milling, melt impregnation	[112]
N-Doped Graphene-Rich Aerogels Decorated with Nickel and Cobalt Nanoparticles	LiBH_4_	melt impregnation	[113]
Edge-Functionalized Graphene Nanoribbon N_2_-cGNR, N_4_-cGNR, and fluorenone GNR (f-cGNR)	Mg(/MgH_2_)	Rieke-like reaction (up to 98% Mg wt.%)	[117]
Ni-Containing Porous Carbon Sheets	LiAlH_4_, NaAlH_4_, and Mg(AlH_4_)_2_	high energy ball-milling	[125]
Nitrogen-Doped Nanoporous Carbon Frameworks	NaAlH_4_	melt infiltration	[128]
Bipyridine-Functionalized MOF (UiO-67bpy)	Mg(BH_4_)_2_	solution infiltration, stirring (DMS dimethyl sulfide solution of Mg(BH_4_)_2_, RT, 2 h)	[151]
Li, Na, and K decorations on 2D honeycomb B_2_O	N/A	theoretical study: dispersion corrected density functional theory (DFT-D2)	[160]
Al_2_O_3_	LiBH_4_-LiI	partial anion substitution in the complex borohydride	[161]
Ni, Cr and Mn/GO	Mg	in-situ reduction Cp_2_Mg, and each transition metal precursor (Cp_2_Ni) dissolved in THF (22.5 mL) added into GO solution, stirred for 30 min. Hydrogen absorption (125 °C, 15 bar H_2_)/desorption (300 °C, 0 bar) Ni-doped rGO–Mg	[163]
Nitrogen doping	Nb	Suppression of nano-hydride growth on Nb(100)	[195]
Pd	Mg NPs; Pd@Mg NPs	Rieke method–co-reduction/precipitation of a Pd^2+^:Mg^2+^ = 1:9 wt. ration (chloride source) in THF, using LiNp as reductant to form Pd@Mg NPs	[196]
Pd/Halloysite Nanotubes (HNTs)	AB (NH_3_BH_3_)	AB encapsulation and thin layer coating of the scaffold Pd/HNTs by solvent infiltration and solvent evaporation (THF) to yield AB@Pd/HNTs. Strong electrostatic adsorption (SEA) of ([Pd(NH_3_)_4_]^2+^) is onto the external surface of HNTs, precursor reduction (H_2_, 250 °C) to form (Pd/HNTs).	[197]

**Table 9:** Examples of recent advances using nanocatalysts to improve kinetic and thermodynamic properties of hydride-based systems in hydrogenation studies.

Hydrogen Storage Class	Hydrogen Storage Material	(Nano)Catalyst Utilized	Ref.
Li-based	LiBH_4_	TiO_2_ (activated carbon nanofibers); N-Doped Graphene-Rich Aerogels Decorated with Ni and Co NPs; Nano-synergy catalyst; Ti_3_C_2_	[108,113,120,185]
	LiAlH_4_	Nickel-Containing Porous Carbon Sheets	[125]
Na-based	NaAlH_4_	Ti; Nickel-Containing Porous Carbon Sheets; Raney Ni; Al; 2D titanium carbide; Ti-based 2D MXene; Two-dimensional C@TiO_2_/Ti_3_C_2_	[82,125,166,167,183,186,188]
Mg-based	Mg NPs, films	Pd; Ti	[196,211,212]
	MgH_2_	VTiCr; catalysts (review); nanocatalysts; anatase TiO_2_; core-shell CoNi@C; TiMn_2_; Carbon scaffold modified by metal (Ni) or non-metal (N); nickel@nitrogen-doped carbon spheres; ultrafine Ni nanoparticles dispersed on porous hollow carbon nanospheres; Nb_2_O_5_ NPs @MOF; Ni/CoMoO_4_ nanorods; Co; (Ti_0.5_V_0.5_)_3_C_2_; ultrafine NbTi nanocrystals, from NbTiC solid-solution MXene; sandwich-like Ti_3_C_2_/TiO_2_(A)-C; FeCo nanosheets; flake Ni nano-catalyst composite; Transition metal (Co, Ni) nanoparticles wrapped with carbon; TiH_2_ thin layer; MgCCo_1.5_Ni_1.5_; MgCNi_3_; supported Co–Ni Nanocatalysts	[34,43,57,60,65,77,92,102,118,147,158,168,184,190,192,201,202,204,205,208,209,210]
	MgB_2_	LiH + TiH_2_	[198]
	Mg(BH_4_)_2_	ultrafine Ni NPs; Ti_3_C_2_; various additives	[132,187,189,207]
	Mg(AlH_4_)_2_	Ni-Containing Porous Carbon Sheets	[125]
Al-based	α-AlH_3_	TiF_3_; Li_3_N	[203,206]
RCH	2LiH + MgB_2_	Ti_3_C_2_	[191]
	2LiBH_4_ − MgH_2_	ZrCl_4_	[134,191]
AB	NH_3_BH_3_	ZIF-67-Derived Co@Porous Carbon; TiO_2_(B) NPs; Pd/Halloysite Nanotubes;	[139,143,197]
Misc.	Pd	Pd@MOF	[68,200]
	B_2_O	Li, Na, and K-Decorated	[160]
	Various hydrides	Alkali/Alkaline Earth Metals; Highly Dispersed Supported Transition Metal; metallic NPs supported on carbon substrates; Heterostructures	[19,20,131,199]
	Carbon aerogel	N-Doped Graphene-Rich Aerogels Decorated with Ni and Co NPs; ZrCl_4_; NEt_3_	[113,134,136]
	Ti_2_N MXene	Pristine (DFT)	[194]

**Table 10:** Hydrogen storage features of nanosized LiH materials.

Additive Used	Other H-Storing Source	H-Storing Composite	wt.% H_2_	Obs.	Ref.
G(graphene)	(LiBH_4_ and LiNH_2_BH_3_ after B_2_H_6_ and BH_3_NH_3_ reaction)	LiH@G (LiH nanospheres, 2 nm thick	6.8 wt.% (50 wt.% LiH in LiH@G); 12.8 wt.% (69.1 wt.% LiBH_4_@G)	LiH@G *T*_onset_ *=* 445 °C, Td ≈ 500 °C (6.8 wt.%). LiNH_2_BH_3_@G *T*_onset_ = 53 °C, 15 °C lower than for bulk LiNH_2_BH_3_; Td ≈ 79 °C. LiBH_4_@G *T*_onset_ *=* 346 °C (124 °C lower than that for bulk), 12.8 wt.%. Li_2_B_12_H_12_ apparent in XRD after 4 cycles (LiBH_4_@G).	[114]
TiCl_4_.2THF	HSAG	LiH@HSAG	1.9 wt.% (340 °C, one step)	Hydrogenation of LiNp(THF) under 0.35 MPa H_2_, 400 rpm, 40 °C, 12 h (cat.:TiCl_4._2THF)	[133]
N/A (TiH_2_)	MgB_2_	LiH/MgB_2_	not investigated	different “top” and “bottom” fractions present in vial. At 700 bar H_2_, 280 °C, 24 h, borohydride formation.	[198]
Activator: hν (light) to Au NPs	N/A (Au)	Au/LiH	11.1 wt.% (as-synthesized); 8.2 wt.% (heat desorption); 3.4 wt.% (light desorption)	plasmonic heating effect of Au NPs (100 °C), under Xe lamp radiation	[214]
LiNH_2_	(Li_3_N)	LiNH_2_ + 2LiH	10.5 wt.%	Li_3_N + 2H_2_ = Li_2_NH + LiH + H_2_ = LiNH_2_ + 2LiH. 2LiNH_2_ = Li_2_NH + NH_3_	[215]
-	-	LiH	12.6 wt.%	LiH = Li + 1/2 H_2_T_m_ = 689 °C; T_d_ = 720 °C	[216]
Si	-	LiH	5 wt.%	Li:Si = 2.35:1; T_d_ = 490 °C	[216]
Co(OH)_2_	-	Li@SiO_2_@Co(OH)_2_	N/A	αLiOH + 2αLi^+^ + 2αe^-^ = α Li_2_O + αLiH (0 < α < 1); High Li^+^ storage in anode	[217]

**Table 11:** Hydrogen storage features of nanosized MgH_2_ -based materials.

Additive/Host Used	Other H-Storing Source	H-Storing Nanocomposite	wt.% H_2_	Obs.	Ref.
4 carbon aerogels, 15 < *D*_avg_ < 26 nm, surface area 800 < *S*_BET_ < 2100 m^2^/g, and total pore volume, 1.3 < *V*_tot_ < 2.5 cm^3^/g	Mg/MgH_2_	MgH_2_@C (MgH_2_ loading: 17–20 vol%, 24–40 wt.%)	3.06 (Mg_CX1); degrades to 1.9 (Mg_CX1, 4th cycle, stable);	Mg(C_4_H_9_)_2_(s) + 2H_2_(g) ! MgH_2_(s) + 2C_4_H_10_(g) 5 cycles des./abs. at 355 °C, 15 h (vacuup/des., 50 bar H_2_/abs.) Mg_CX1, Mg_CX2 – 0.046 wt.%H_2_/min (best result in this study). Highlights the importance of conducting des/abs cycles (different results obtained for “conditions 1 and 2”).	[53]
Mg-B	Mg-B/MgH_2_/ MgB_12_H_12_/Mg(BH_4_)_2_	Mg-B (MgB_0.75_)	N/A (abs., 280 °C, 700 bar H_2_, MgB_0.75_), N/A (abs., 380 °C, 700 bar H_2_, MgB_2_),	nanoscale Mg–B material (MgB_0.75_) made by surfactant ball milling MgB_2_ in a mixture of heptane, oleic acid, and oleylamine	[55]
core-shell CoNi@C	CoNi: 2 coupled H-pumps: Mg_2_Co/Mg_2_CoH_5_ and Mg_2_Ni/Mg_2_NiH_4_,	MgH_2_-8 wt.% CoNi@C	5.83 (275 °C, 1800 s); 6.17 (300 °C, 1800 s); 6 (150 °C, 200 s)	173 °C dehydrogenation onset for MgH_2_-8 wt.% CoNi@C. Excellent thermal conductivity of the nanocomposite due to C-shell. E_a, des_ = 78.5 kJ mol^−1^.	[65]
TiMn_2_	Mg/MgH_2_	MgH_2_/10 wt.% TiMn_2_	5.1 (reversible, 225 °C, 100 s, 10 barH_2_/abs; 400 s, 0.2 bar H_2_/des.)	cold pressing technique; potential for PEM fuel cell applications. E_a,des_ = 82.9 kJ mol^−1^; E_a,abs_ = 19.3 kJ mol^−1^. 414 cycles within 600 h continuously without degradations (hydrogen flow at an average rate of 150 mL/min)	[77]
Ni	Ni_4_B_3_ intermediate confirmed by XRNES	Ni-doped-2LiBH_4_–MgH_2_ in graphene	0.47 (0.48 theoretical)	ball milling 2LiBH_4_-MgH_2_-Ni/C (x = 0, 5, 10, 15). Heterogeneous nucleation of MgNi_3_B_2_. X-ray absorption near-edge structure (XRNES) used to probe intermediate Ni_4_B_3_ 3LiBH_4_ + 4Ni = 3LiH + Ni_4_B_3_ + 4.5H_2_	[78]
CoS nano-boxes scaffold	Mg/MgH_2_; MgS-catalytic effect	MgH_2_@CoS-NBs	3.17 (300 °C); 3.37 (400 °C)	hydriding and dehydriding enthalpies (−65.6 ± 1.1 and 68.1 ± 1.4 kJ mol^−1^ H_2_. hydriding and dehydriding (57.4 ± 2.2 and 120.8 ± 3.2 kJ mol^−1^ H_2_)	[80]
Ni- or N-doped C scaffold: xNi-CMK-3 (x = 1 and 5 wt.%) and N-CMK-3	Ni	MgH_2_@xNi-CMK-3; MgH_2_@ N-CMK-3	7.5 (MgH_2_@5Ni-CMK-3); 6.5 (MgH_2_@1Ni-CMK-3 and MgH_2_@N-CMK-3) at 200 °C, 2 h	Hydrogenation is faster at 300 °C, MgH_2_@5Ni-CMK-3, MgH_2_@1Ni-CMK-3 and MgH_2_@N-CMK-3 absorb 6 wt.% H_2_ in 10 min (6.5 wt.%, 2 h). Enhanced kinetics, Ea: MgH_2_@CMK-3 (125.3 ± 2.1 kJ mol^−1^); MgH_2_@N-CMK-3 (116.2 ± 1.8 kJ mol^−1^), MgH_2_@1Ni-CMK-3 (109.2 ± 1.3 kJ mol^−1^), MgH_2_@5Ni-CMK-3 (107.6 ± 1.2 kJ mol^−1^)	[92]
Mg-TiC_X_@C	TiC_x_	Mg-TiC_X_@C	4.5 (des., 60 min, 300 °C); 5.5 (abs., 25 min, 250 °C)	TiC_X_-decorated Mg nanoparticles (NPs) in 2–3 nm carbon shells through a reactive gas evaporation method. Mg_88_(TiC_0.6_)_12_@C best results. Stable after 10 hydrogenation/dehydrogenation cycles at 250/300 °C.	[94]
Monodispersed single-crystal-like TiO_2_ with amorphous carbon	-	Mg@C-TiO_2_	6.5 (des. 275 °C, 10 min.); 6.5 (abs., 200 °C, 5 min)	reductions in hydrogen desorption temperature (163.5 °C) and E_a_ (69.2 kJ mol^−1^). The sample can be fully rehydrogenated with a reversible capacity of 6.5 wt.% at 200 °C within 5 min.	[95]
Graphene nanosheet (GN)	-	MgH_2_@GN-40wt.%	4.5 (reversible, 6 cycles, 300 °C)	E_a_ = 80.8 kJ mol^−1^ (des., 0.01 atm H_2_) MgH_2_ size tunable by adjusting MgBu_2_/G wt. ratio before hydrogenation	[98]
nickel@nitrogen-doped carbon spheres	Ni/Mg_2_NiH_4_	MgH_2_–Ni@NCS	4.3 (des.), 5.7 (abs.) in 8 min, 350 °C; 4.2 (abs., 60 min, 100 °C)	high-energy ball milling process; negligible degradation after 10 cycles. In situ formed Mg_2_NiH_4_ induced dehydrogenation of MgH_2_ and prevented Mg agglomeration.	[102]
AlH_3_@CNT	AlH_3_	MgH_2_/AlH_3_@CNT	8.20 (des., 1 h, 200 °C); 5.61 (abs., 0.16 h, 250 °C)	CNTs: high specific surface area (550 m^2^ g^−1^), small diameter (6–8 nm), afford 60–80 nm crystal size nanocomposite MgH_2_/AlH_3_@CNT nanoparticles, releases H_2_ at ~71 °C.	[109]
Graphene Nanosheet GNS	-	MgH_2_ –10 wt.% GNS	5.1 (des., 20 min, 325 °C); 5.2 (abs., 10 min, 250 °C)	well-dispersed MgH_2_ nanoparticles (~3 nm); confinement effect of graphene	[115]
ultrafine Ni nanoparticles dispersed on porous hollow carbon nanospheres	Mg_2_Ni/Mg_2_NiH_4_	Ni loading up to 90 wt.% in composite catalyst; MgH_2_-5 wt.% (90 wt.%Ni-10 wt.%CNS)	6.4 (reversible). 6.2 (des. 30 min, 250 °C; abs., 250 s, 150 °C)	Des. onset (190 °C) and des. peak (242 °C). Reversible capacity of 6.4 wt.% achieves after 50 cycles at a moderate cyclic regime.	[118]
Graphene oxide (GO), reduced graphene oxide (rGO)	-	MgH_2_@GO, MgH_2_@rGO (rGO50, rGO100, and rGO200)	6.25 (200 °C, 15 bar H_2_, MgH_2_@GO)	role of graphene defects; rGO is detrimental, as Ea is lower on defected GO. MgH_2_@rGO: disturbed diffusion pathway of hydrogen atoms caused by the coalesced morphology	[121]
Reduced graphene oxide (rGO)	Mg/MgH_2_	Mg/rGO	6.2 (des., 2 h, Mg{21̅1̅6}) 5.1 (des, 2 h, (random)Mg/rGO)	preferential orientation of Mg/rGA nanocomposites was investigated: Mg growth on {0001} and {21̅1̅6} planes of rGO Mg (21̅1̅6) stabilizes hydrogen absorption thermodynamics	[122]
1D Carbon Matrix, fishbone shaped (CNF)	-	Ultrathin Mg Nanosheet @ 1D-C	6 (abs., 1 h, no catalyst, 200–250 °C); 6 (des, 1.5 h, 200–325 °C)	90% of the total capacity is absorbed within 1 h at all temperatures and desorbed within 1.5 h	[124]
AC activated carbon	LiBH_4_	2LiBH_4_-MgH_2_ @AC (LB-MH-AC)	5.7 (theoretical); 2.56–4.55 (350 °C, abs. under 30–40 bar H_2_)	melt infiltration of hydride in AC (400 °C, 40–50 bar H_2_, 10 h) improvement of thermal conductivity of materials and temperature control system could alleviate wt.% decrease	[130]
ZrCl_4_- doped carbon aerogel scaffold (CAS)	2LiBH_4_–MgH_2_	2LiBH_4_–MgH_2_@ ZrCl_4_-CAS x wt.% (x = 50, 67, 75)	5.4 (5.7, theoretical, x = 50); 3.4 (3.8 th., x = 67); 2.5 (2.9 th., x = 75) at 301–337 °C	melt infiltration technique. Up to 97 and 93% of theoretical H_2_ capacity released and reproduced, respectively. 2LiBH_4_ + MgH_2_ = 2LiH + MgB_2_ + 4H_2_ (350–500 °C)	[134]
3-D activated carbon with TM dispersion (Co, Fe, and Ni)	TM/(TM)H_x_	MgH_2_@3D-AC (MHCH)	6.63 (abs., 5 min, 180 °C, for Ni-MHCH-5); 6.55 (des., 75 min, 180 °C)	TEA ((HOCH_2_CH_2_)_3_N)/NH_2_NH_2_ reduction in ^n^Bu_2_Mg-infiltrated 3D-C. MgH_2_ embedded in 3D-AC with periodic synchronization of transition metals (MHCH). Excellent long-term cycling stability over ~435 h for MHCH-5. Ni more efficient than Co or Fe.	[137]
nano-TiO_2_@C	Mg/MgH_2_	MgH_2_-10 wt.% TiO_2_@C	6.5 (7 min, 300 °C, des.); 6.6 (10 min, 140 °C, abs.)	10 wt.% nanocrystalline TiO_2_@C weakens the Mg-H bond, thus lowering desorption temperature	[146]
Nb_2_O_5_@MOF	Nb_2_O_5_@MOF	7 wt.% Nb_2_O_5_@ MOF doped MgH_2_	6.2 (6.3 min, 250 °C; 2.6 min, 275 °C)	Desorption onset: 181.9 °C. E_a_ = 75.57 ± 4.16 kJ mol^−1^ Absorption: 4.9 wt.% (6 min, 175 °C); 6.5 wt.% (6 min, 150 °C); E_a_ = 51.38 ± 1.09 kJ mol^−1^ Capacity loss: 0.5 wt.% after 30 cycles	[147]
Ni-MOF (7.58 nm, 0.46 cm^3^g^−1^)	Mg_2_Ni/Mg_2_NiH_4_	MgH_2_@Ni-MOF	4.03 abs-3.94 des (325 °C); 4.02 abs-3.91 des (350 °C); 3.95 abs = 3.87 des (375 °C). The Ni-MOF contribution (physisorption): 0.91 (325 °C); 0.85 (350 °C), 0.97 (375 °C), 0.88 (300 °C).	The abs/des plateau pressure: 4.63 atm/3.45 atm (325 °C). thermodynamics (−65.7 ± 2.1 and 69.7 ± 2.7 kJ mol^–1^ H_2_ for ab-/desorption, respectively) and kinetics (41.5 ± 3.7 and 144.7 ± 7.8 kJ mol^−1^ H_2_ for ab-/desorption, respectively) of Mg/MgH_2_ in the MgH_2_@Ni-MOF composite. The Ni-MOF scaffold acts as “aggregation blocker”. shortened H diffusion distance results in the ultrafast H diffusion rate in the nanosized Mg/MgH_2_. (C_4_H_9_)_2_Mg + 2H_2_ → MgH_2_ + 2C_4_H_10_ (g) 2(C_4_H_9_)_2_Mg + Ni(Ni-MOF) + 4H_2_ → Mg_2_NiH_4_ + 4C_4_H_10_ (g) Mg_2_NiH_4_ = Mg_2_Ni + 2H_2_ (g)	[149]
(Ni/Co)MoO_4_ nanorods	Mo/Mg_2_Ni/Mg_2_NiH_4_	MgH_2_-10 wt.% NiMoO_4_ MgH_2_-10 wt.% CoMoO_4_	7.41 (319.4 °C, MgH_2_) 6.51 (243.3 °C, MgH_2_-10 wt.% NiMoO_4_) 6.49 (277.6 °C, MgH_2_-10 wt.% Co-MoO_4_) from TPD up to 400 °C, 3°/min. 6 (des., MgH_2_-NiMoO_4_, 10min, 300 °C) 5.5 (abs., MgH_2_-NiMoO_4_, 10 min, 300 °C, 31.6 atm H_2_)	Ni/CoMoO_4_ were doped into MgH_2_ ball milling method at 400 rpm with a ball-to-powder ratio of 60:1 for 6 h. superior promoting effect of NiMoO_4_ over CoMoO_4_; NiMoO_4_ reacts with MgH_2_ during the first dehydrogenation to in situ form Mg_2_Ni and Mo^0^, Mg_2_Ni/Mg_2_NiH_4′_ mutual transformation upon hydrogen release/uptake is the well-known ‘hydrogen pump’. Mo^0^ played for the hydrogen storage in MgH_2_: (i) it accelerates the hydrogen de/absorption of MgH_2_ through weakening the Mg–H bonding; (ii) it facilitates the mutual ‘Mg_2_Ni/Mg_2_NiH_4′_. No tdn effect: ΔHabs./ΔHdes of −71.14/78.25 close to that of pure MgH_2_: −72.42/74.08 kJ mol^−1^	[158]
Co	Mg_2_CoH_5_ and Mg_6_Co_2_H_11_	2MgH_2_-Co	4.43 (pellet); 2.32 (powder)	high pressure compacting in pellet doubles H_2_ storage	[168]
ScH_2_, YH_3_, TiH_2_, ZrH_2_, VH and NbH	Mg/MgH_2_	0.95 MgH_2_–0.05 (TM)H_x_	≥5 wt.%	(TM)H_x_ crystallite size of ∼10 nm, obtained by mechanochemistry (RMB, reactive ball milling) MgH_2_ + TM (Sc, Y, Ti, Zr, V, Nb) under H_2_ pressure. Early Transition Metals (ETM) chosen by the known stability of their respective hydrides under normal conditions.	[169]
BiphasicMgH_2/_TiH_2_ within Mg–Ti–H NP	Mg/MgH_2_	Mg–x at.%Ti–H NPs Mg-14Ti-H and Mg-63Ti-H (26–10 nm)	4 (x = 7); 2.2 (x = 35); 0.8 (x = 63) abs, full at 150 °C.	Equilibrium data for H_2_ ab-/de-sorption by Mg/MgH_2_ at low 100–150 °C range. Fast H_2_ release from MgH_2_ at 100–150 °C (no Pd catalyst). The free energy change at the TiH_2_/Mg interface induces MgH_2_ destabilization. Hydrogen uptake (100 s) and release (1000 s, 0.1…0.2 wt.%/min) for Mg–x at.%Ti–H NPs.	[170]
TiO_2_ (anatase)	TiO_2_/Mg	MgH_2_-TiO_2_	2.70 (abs, 500 s, 100 °C.); 4.5 (abs, 100 °C. 120 min); 5.3 (abs., 44 s, 200 °C) for MgH_2_-5 wt.% TF70:	Influence of TiO_2_ facets {001} and {101}: MgH_2_-TiO_2_{001} superior properties. E_a,des_ = 76.1± 1.6 kJ mol^−1^ for MgH_2_-TF70	[172]
Multilayer Ti_3_C_2_ (ML-Ti_3_C_2_)	Ti_3_C_2_	MgH_2_ + *x* wt.% ML-Ti_3_C_2_, *x* = 4, 6, 8, 10	6.45 (des.; 240 °C, 10 min.) 1.95–3.63 (des.; 140 °C, in 10–60 min). 6.47 (abs. 150 °C); 4.20 (abs., 75 °C)	Ti_3_C_2_ was introduced into MgH_2_ by ball milling. ML-Ti_3_C_2_ prepared in-house, by chemical exfoliation.	[177]
Ti_3_C_2_	Mg/MgH_2_	MgH_2_-*x* wt.% Ti_3_C_2_ (*x* = 0, 1, 3, 5 and 7)	6.2 (x = 5; 1 min, 300 °C, des.); 6.1 (x = 5; 30 s, 150 °C)	MgH_2_-5 wt.% Ti_3_C_2_ shows excellent dehydrogenation/hydrogenation kinetics (chargind/discharging in <1 min)	[193]
Pd	Pd/PdH_x_; Mg-Mg_6_Pd	Mg@Pd: γ-MgH_2_, PdH_0.706_	3 (abs, 50 °C, 2 h)	Mg NPs (40–70 nm). E_a,des_ = 93.8 kJ/mol at 216.8 °C; E_a,des_ = 44.3 kJ/mol at 50 °C. ΔH_des_ = 72.7 kJ/mol; ΔH_abs_ = −71.5 kJ/mol. Pd-Mg alloy important role.	[196]
FeCo nanosheets	FeCo (50nm)	FeCo-catalyzed MgH_2_	6 (des., 9.5 min, 300 °C), 6.7 (abs, 1 min, 300 °C); 3.5 (abs, 10 min, 150 °C)	E_a,des_ = 65.3 ± 4.7 kJ mol^−1^ (60 kJ mol^−1^ reduction from pristine MgH_2_) E_a,abs_ = 53.4 ± 1.0 kJ mol^−1^	[201]
flake Ni nano-catalyst composite	Mg_2_Ni/Mg_2_NiH_4_	MgH_2_ + 5 wt.% Ni	6.7 (des., 3 min, 300 °C). 4.6 (125 °C, 20 min, 29.6 atm H_2_)	E_a, des_ = 71 kJ mol^−1^; E_a,abs_ = 28.03 kJ mol^−1^.	[202]
TM (Co, Ni) nanoparticles wrapped with carbon	Mg_2_Ni/Mg_2_NiH_4_	MgH_2_-6%Ni/C	6.1 des. at 250 °C; 5.0 (abs., 100 °C, 20 s)	dehydrogenation temperature 275.7 °C. Absorption/desorption stability with respect to both capacity (up to 6.5 wt.%) and kinetics (within 8 min at 275 °C for dehydrogenation and within 10 s at 200 °C for rehydrogenation	[204]
TiH_2_	-	MgH_2_-TiH_2_	6.45 (DFT)	MgH_2_/TiH_2_ interface is thermodynamically stable, and promotes the generation and diffusion of hydrogen.	[205]
MgCCo_1.5_Ni_1.5_	Mg_2_NiH_4_, MgC_0.5_Co_3_ and C catalysts (from MgCCo_1.5_Ni_1.5_)	Mg/MgH_2_-MgCCo_1.5_Ni_1.5_	6.1–6 (abs, 5 min, 350 °C, 1st cycle-10th cycle); 5.9-5.8 (des, 1st cycle-10th cycle)	ball-milling and hydriding combustion method. Desorbs H_2_ at 216 °C (onset). E_a,des_ = 39.6 kJ mol^−1^	[208]
MgCNi_3_	MgCNi_3_	MgH_2_-MgCNi_3_	4.42 (abs, 150 °C, 1200s)	Mg-MgCNi_3_ composite shows excellent cyclic stability with a 98% retention rate.	[209]
Co–Ni Nanocatalysts	Mg/MgH_2_	0.95MgH_2_–0.5(CoNi(OH)*_x_*); Ni@G-doped MgH_2_; CoNi@G-doped MgH_2_	6.5 (des, Ni@G-doped MgH_2_, 45 min, 260 °C; or 25 min, 280 °C)	ball milling MgH_2_ and Co-Ni, 5 bar H_2_, 2 h, 400 rpm, 20:1 BPR. Co-subst. of Ni changes shape of catalyst (sphere-to-plate) and decreases catalytic efficiency.	[210]
Mg-Ti-H nanoparticles	Mg/MgH_2_	Mg-x Ti-H NPs (x = 14…63 at.%)	4.2 (22 at.%Ti, at 100…150 °C)	gas phase condensation of mixed Mg-Ti vapors under H_2_. E_a,abs._ 43…52 kJ/mol, the rate constant (150 °C) increases from 2.7×10^−2^ s^−1^ to 9.2×10^−2^ s^−1^ with increasing [Ti]. Hydrogen desorption: sequence of surface-limited (E_a_ = 32 kJ/mol) and contracting-volume kinetics, except at the highest Ti content where nucleation and growth is observed. k_des_ (at 150 °C) increases from 0.5–10^−3^ s^−1^ to 1.2×10^−3^ s^−1^ with [Ti]. The activation energy for H_2_ recombination is remarkably small (~32 kJ/mol)	[211]
light activation, Au(HAuCl_4_)	-	Mg@Au, hν	5.2 (350 °C, 3h, Mg_bulk_); 4.9 (350 °C, 3 h, MgH_2_-Au 5 wt.%)	Rehydrogenation at 12 h illumination, 14.8 atm H_2_. No H_2_ release at 100 °C, limited at 200 °C (0.7 wt.%) and at 300 °C (1wt.%) for Mg@Au 5 wt.%.	[214]
-	-	ultrafine MgH_2_	6.7 (reversible; abs: 360 min, 30 °C; or 60 min, 85 °C, 30 bar H_2_); vol capacity: 65.6 gH_2_/L	novel metathesis process of liquid–solid phase driven by ultrasonication (2 h) was proposed from THF. Pressed into pellet under 200 MPa.Stable and rapid hydrogen cycling behavior in 50 cycles at 150 °C. Equilibrium pressure: 0.0304 (120 °C), 0.151 (160 °C), 1.014 (215 °C), 30-20-10 times higher than that of pristine MgH_2_.	[222]
Mg(B_3_H_8_)_2_	Mg(B_3_H_8_)_2_	Mg(B_3_H_8_)_2_-MgH_2_	2.16 (93.6…138 °C)	Synergistic role in Mg(B_3_H_8_)_2_-MgH_2_ composite. No H_2_ release below 150° for the pristine components.	[224]

**Table 12:** Hydrogen storage features of nanosized AlH_3_ materials.

Additive Used	Other H-Storing Source	H-Storing Composite	wt.% H_2_	Obs.	Ref.
Al-doping (925 ppm), AC	AC@MOF (activated carbon@MOF)	Al@AC-MIL-101	0.55 (MIL-101)…1.74 (Al@AC-MIL-101)	Al/α-AlH_3_ hydrogenation release/uptake cycles ran at 298 K, and pressure < 100 bar H_2_	[40]
HSAG (high surface area graphite)	-	AlH_3_@HSAG	0.25 (14.4 wt.%AlH_3_ by ICP-OES data, and only 15% of Al behaves reversibly)	H_2_ uptake commences at 60…270 °C (mean 150 °C, peak 165 °C) and 60 bar H_2_ pressure	[44]
CTF-bipyridine (CTF-bipy) AlH3@CTF-	-	AlH_3_@CTF-bipyridine; AlH_3_@CTF-biph—no reversibility (bipyridyl group was compulsory)	0.65, 0.58, 0.57 (2nd, 3rd, 4th cycles)	H_2_ desorption between 95…154 °C rapidly (completes at 250 °C) from AlH_3_@CTF-bipyridine composite. Reversible at 700 bar H_2_ and 60 °C (incomplete, 24 h).	[51]
CNT	MgH_2_	MgH_2_/AlH_3_@CNT	8.2 (1 h, 200 °C, dehydrogenation); 5.61 (0.16 h, 250 °C)	CNT prevent aggregation and enhance MgH_2_-ALH_3_ interaction	[109]
TiF_3_	-	α-AlH_3_/LiCl-TiF_3_ (3:1:0.1 molar ratio)	9.92 (80–160 °C, 750 s)	α-AlH_3_ obtained by milling of LiH and AlCl_3_.	[203]
Li_3_N	Li_3_N	Li_3_N@AlH_3_	8.24 (100 °C); 6.18 (90 °C) and 5.75 (80 °C)	10 wt.% doping of AlH_3_ with Li_3_N leads to ~8.0 wt.% H_2_ release at 100 °C. Nanoscale dispersion of the two hexagonal phases (AlH_3_, Li_3_N) by ball milling. E_a_ = 100.4 kL mol^−1^ (0.9 AlH_3_-0.1 Li_3_N)	[206]
-	-	AlH_3_	10.0 (140 °C, 3600 s)	neat AlH_3_ (commercial)	[227]
Al(OH)_3_	-	core-shell α-AlH_3_@Al(OH)_3_	10.0 (140 °C, 1000 s)	α-AlH_3_@Al(OH)_3_ nanocomposite can be stored in air (7 days)	[227]

**Table 13:** Hydrogen storage features of nanosized (TM) H_x_ materials.

Additive Used	Other H-Storing Souce	H-Storing Composite	wt.% H_2_	Obs.	Ref.
porous carbon (HSAG)	Co	Mg_2_CoH_5_@HSAG Co + MgH_2_@HSAG	N/A	bottom-up approach affords PSD 2–50 nm (max. at 15 nm); wt.% H_2_ capacity decrease due to Mg oxidation. 2MgH_2_ + Co + ½ H_2_ → Mg_2_CoH_5_ MgH_2_ NPs: 5nm. Co@Mg NPs: 7 nm. Desorption temperature increases with cycle number (1..7) in a 5 wt.% Mg_2_CoH_5_@HSAG nanocomposite from ~590 to ~610 K. Possible disproportionation: Mg_2_CoH_5_ → MgH_2_ + Mg_6_Co_2_H_11_	[97]
ScH_2_, YH_3_, TiH_2_, ZrH_2_, VH and NbH	MgH_2_	0.95 MgH_2_–0.05 (TM)H_x_	≥5 wt.%	(TM)H_x_ crystallite size of ~10 nm, obtained by mechanochemistry (RMB, reactive ball milling) MgH_2_ + TM (Sc, Y, Ti, Zr, V, Nb) under H_2_ pressure. Early Transition Metals (ETM) chosen by the known stability of their respective hydrides under normal conditions.	[169]
MOF	Ir, Rh	TM-H@MOF (TM = Pd, Ir, Rh)	0.18H/Pd (cubes); 0.27H/Pd (octahedrons)	ΔH_abs_ of the TM NPs change from endothermic to exothermic with decreasing particle size. Pd@HKUST-1 [copper(II) 1,3,5-benzenetricarboxylate (Cu_3_(BTC)_2_] abs. 0.87H/Pd compared to Pd(bulk, cubes, 0.5 H/Pd)	[200,234]
Mg nanofilm	Mg	Pd NPs@Mg film	N/A	*D*_H_^film^ ≈ 8 × 10^−18^ m^2^ s^−1^	[212]
-	-	TaH_x_ (0 < x < 0.7)	<0.389 wt.%	higher H-sensing activity than Pd-alloy (10^−2^…10^+4^ Pa H_2_)	[216]

## Data Availability

Not applicable.
